# A Quantitative Printability Framework for Programmable Assembly of Pre‐Vascular Patterns via Laser‐Induced Forward Transfer

**DOI:** 10.1002/adhm.202503665

**Published:** 2025-11-21

**Authors:** Cécile Bosmans, Núria Ginés Rodriguez, Ulisses Jesús Gutiérrez Hernández, David Fernandez Rivas, Marcel Karperien, Jos Malda, Liliana Moreira Teixeira, Riccardo Levato, Jeroen Leijten

**Affiliations:** ^1^ Department of BioEngineering Technologies Faculty of Science and Technology TechMed Centre University of Twente Enschede The Netherlands; ^2^ Department of Orthopaedics University Medical Center Utrecht Utrecht The Netherlands; ^3^ Regenerative Medicine Center Utrecht Utrecht The Netherlands; ^4^ Mesoscale Chemical Systems group University of Twente Enschede The Netherlands; ^5^ Department of Clinical Sciences Faculty of Veterinary Medicine Utrecht University Utrecht The Netherlands; ^6^ Present address: Departamento de Física Facultad de Ciencias Universidad Nacional Autónoma de México Mexico

**Keywords:** additive manufacturing, biofabrication, in vitro models, micropatterning, vasculogenesis

## Abstract

The defined vascularization of complex and intricate tissue constructs remains an unmet need in tissue engineering and regenerative medicine. While large constructs require vasculature for oxygen, nutrient supply, and waste clearance, their incorporation within biofabricated tissues is essential for developmental and disease modeling studies. There is, therefore, a critical demand to establish reproducible and organized vascular networks within in vitro models to ensure experimental robustness and quantitative interpretability. Current micropatterning and biofabrication strategies are limited in emulating native geometrical complexity, throughput, and resolution, while self‐assembly approaches rely on inherently random network formation. Here, laser‐induced forward transfer (LIFT) is utilized, offering high spatial resolution for deterministic micropatterning of cells with high viability. A unique droplet quality assessment framework is established through a multiparametric study to objectively identify a printability window, assigning a single‐indexed score per printing condition. Within the optimal transfer regime, control over droplet concentration is demonstrated. The impact of pattern density on early vascular morphogenesis is explored, highlighting the effect of geometrical design on network formation. Finally, these findings are leveraged for the spatially controlled assembly of multicellular vascular patterns, offering a reproducible strategy for high‐resolution micropatterning and addressing a key limitation in the biofabrication of physiologically relevant tissue models.

## Graphical abstract

Laser‐Induced Forward Transfer (LIFT) is presented as a powerful micropatterning tool. An objective printability framework is developed to assess optimal printing parameter combinations. The technology is further explored for its ability to deterministically deposit microdroplets at predefined locations following CAD designs, enabling the patterning of vascular‐like hierarchical branching structures.

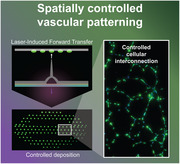

## 1 Introduction

The vascularization of critical‐sized tissue constructs remains a major challenge in the field of regenerative medicine. The incorporation of spatially defined vascular features is essential for studying cell interactions, developmental processes, and drug responses in engineered tissues and organ‐on‐chip systems [1]. In particular, the patterning of vascular networks has been shown to aid the diffusion of nutrients and oxygen toward larger tissue constructs [2]. However, generating reproducible and well‐organized vascular‐like networks remains a key challenge in reducing the variability and unpredictability of culture models [3]. Achieving structural control is also essential to enable quantitative interpretation. Therefore, strategies that enable deterministic spatial patterning are highly beneficial in addressing this unmet need.

Diverse microfabrication and biofabrication techniques have been explored to spatially pattern vascular features onto 2D cell‐laden constructs or acellular hydrogel substrates. Microfabrication approaches, such as photolithography and soft lithography, enable the precise molding of microvascular patterns, often using elastomeric stamps or photomasks to cast materials like polydimethylsiloxane (PDMS) or bioactive materials such as hydrogels and bioinks [4, 5]. These methods offer high reproducibility, but are generally limited to single‐layer geometries, with complex patterning requiring time‐intensive manipulation, such as alignment between structures and stacking of different substrates [6, 7]. Laser‐based ablation and two‐photon polymerization can achieve contactless, freeform patterning with high‐resolution structuring of bioinks [8]. Nevertheless, these technologies face significant limitations in throughput, system accessibility, material compatibility, and cell viability.

Biofabrication technologies facilitate the fabrication of complex, multi‐layered constructs and surface micropatterning in a rapid and reproducible manner. Several enabling technologies allow precise deposition of materials and cells in three dimensions, including extrusion, inkjet, laser‐induced forward transfer (LIFT), and stereolithography [9]. In brief, extrusion bioprinting has been extensively used to print sacrificial hydrogels and vascular structures [10]. Stereolithography (SLA) has been widely applied to automatically produce layer‐by‐layer constructs with high freedom of design [11]. Although these methods allow for the patterning of large‐scale tissue constructs, their resolution remains low compared to the intricate structures and physiological dimensions of small blood vessels and capillaries.

Other extensively explored avenues in the engineering of microvessels rely on cellular self‐assembly. The combination of endothelial cells with supporting cell types in 3D environments has been shown to result in cellular self‐organization into microcapillary networks containing hollow lumens [12, 13]. While this approach allows reproduction of the cellular organization found in microvessels, it lacks the hierarchical stratification of vessel branching. Consequently, there has been increasing interest in combining the use of micropatterning technologies with cellular self‐assembly [14]. These strategies aim to combine topological cues and local delivery of cells and materials [15]. Moreover, micropatterned vessel‐like structures are anticipated to guide deterministically the self‐assembly process into predictable and reproducible, highly complex, hierarchically branched vascular structures.

LIFT is a nozzle‐free bioprinting technique that uses laser pulses to direct‐write picoliter droplets from a donor layer onto a receiver substrate. It allows for contactless, precise deposition of patterns across a wide range of viscosities and cell types with high post‐printing viability [9]. LIFT has therefore shown promise for tissue engineering applications, including microvascular patterning. Prior studies have reported printing endothelial cells onto preformed stromal or osseous substrates to guide capillary‐like self‐assembly, often relying on layer stacking or living biopaper approaches [16, 17]. While promising, such methods typically depend on fixed substrate architectures and context‐specific configurations. This limits their adaptability to broader biofabrication workflows that require tunable hydrogel environments or dynamic culture platforms such as open‐top organ‐on‐chip systems. The direct micropatterning of multicellular structures with precise spatial control on soft, reconfigurable substrates, therefore, remains underexplored. Addressing this gap is critical for expanding the use of LIFT toward versatile and reproducible vascular patterning applicable across tissue models.

Here, we introduce LIFT as a robust, high‐resolution bioprinting approach to directly micropattern endothelial and supporting cells with precise spatial control on soft hydrogel substrates. We systematically mapped key LIFT parameters across a broad range of ink formulations to define a reproducible printability window. To quantify printing performance, we developed an integrated printability score combining droplet diameter, transfer efficiency, and satellite droplet formation. This framework enables predictive optimization of printing conditions across various viscosities and offers a potential route for standardizing print quality in future LIFT applications. Moreover, we demonstrated that cell patterning followed a Poisson distribution, enabling deterministic micropatterning of cellular constructs. High post‐printing viability was maintained for both human umbilical vein endothelial cells (HUVEC) and bone marrow‐derived mesenchymal stromal cells (BM‐hMSC) by optimizing laser energy, loading volume, and substrate composition. With our gained insights, we printed endothelial‐like patterns at varied cell and droplet concentrations and inter‐droplet distances, revealing the influence of these variables on early vascular morphogenesis. Finally, we utilized LIFT to micropattern direct co‐cultures of HUVEC and BM‐hMSC, tuning receiver substrate materials to promote the formation of microvascular‐like networks and maintain pattern integrity over a 5‐day culture period. Altogether, these findings position LIFT as a versatile tool for spatially controlled microvascular‐like biofabrication and lay the foundation for predictive multicellular patterning across diverse culture systems.

## 2 Results and Discussion

### 2.1 LIFT Mechanism and Jetting Dynamics

**FIGURE 1:**
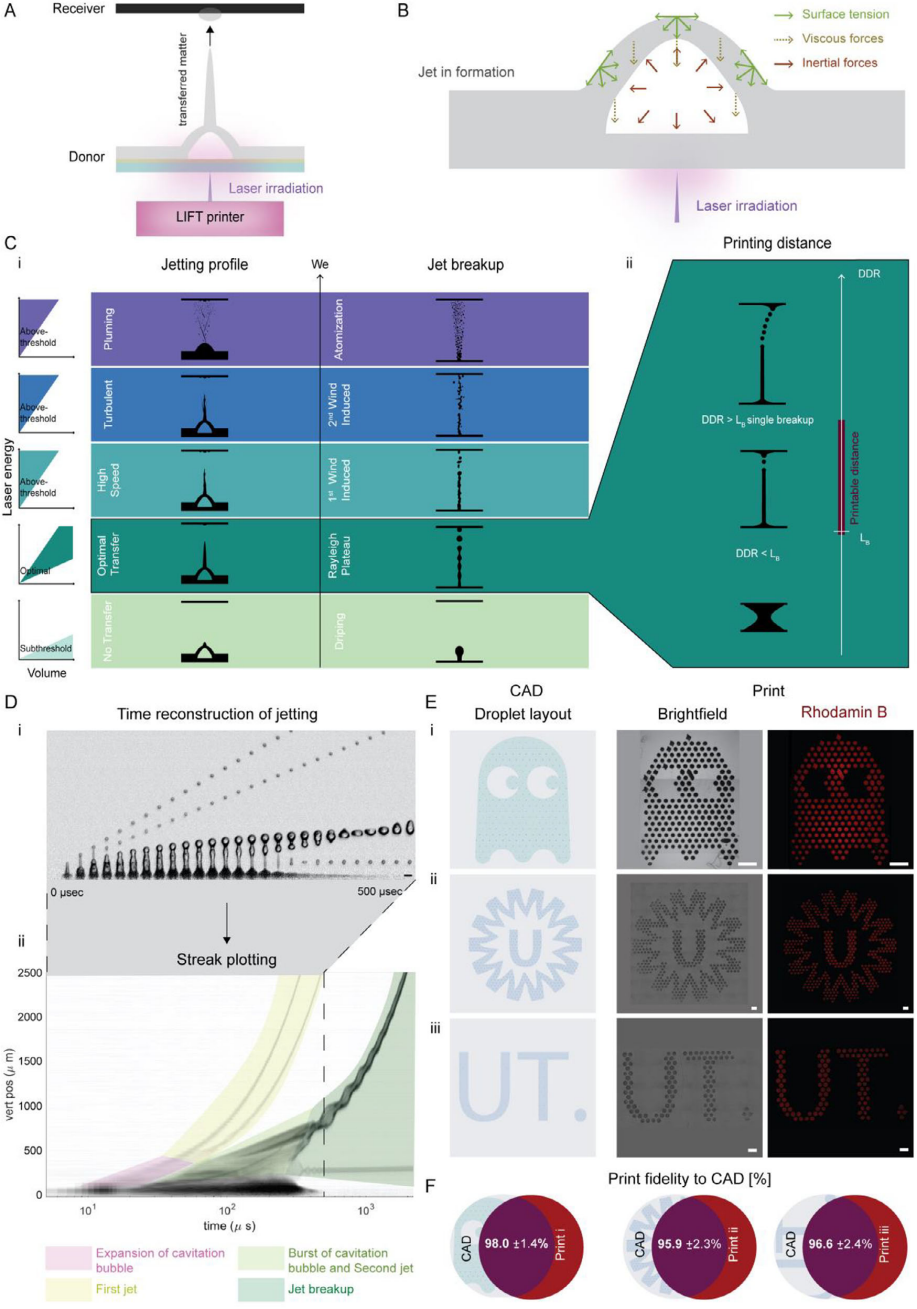
LIFT mechanism, jetting dynamics, and patterning capabilities. (A) Schematic depiction of the LIFT setup, where a laser beam is focused on a donor substrate composed of a glass coated with a Ti/Au bilayer, onto which a thin film of ink is manually deposited. Upon irradiation of the metallic layer, a jet transfers the matter to the receiver placed forward. (B) Schematic representation of the forces governing jet formation during LIFT and their direction, including surface tension, viscous, and inertial forces. (C) (i) Jetting regimes and their breakup profiles as a function of the Weber number (inertial to surface tension force ratio), revealing subthreshold, optimal, and above‐threshold transfer zones, essentially governed by laser energy and donor ink film volume. (ii) impact of transfer distance relative to the breakup length of a jet, determining the accuracy of deposition and jetting transfer as opposed to contact‐based transfer. DDR: Distance donor receiver; Lb: Length breakup. (D) Optimal transfer is further illustrated by (i) time‐resolved high‐speed imaging of a jet at 5 µJ and (ii) its corresponding streak plot (logarithmic time scale). Key events of the printing process are highlighted, namely the expansion of the cavitation bubble initiating the first jet, followed by bubble collapse and subsequent second jet formation, which breaks into droplets. (E) (i‐iii) Example of CAD and corresponding printed patterns, imaged via brightfield or fluorescence microscopy. CAD: Computer‐aided design. (F) Print fidelity, quantified as the deviation between target and actual inter‐droplet distances. Analysis based on n = 50 inter‐droplets per N= 1 print. All scale bars indicate 500 µm.

The core structure and jetting mechanism of our LIFT bioprinting platform are schematically illustrated in Figure 1A. LIFT relies on a spatially modulated pulsed laser targeting a bioink layer to generate a cavitation bubble that propels a jet of ink onto a receiver substrate [18]. Inertial forces accelerate the ink forward, while surface tension and viscous forces resist this motion (Figure 1B). These contributions collectively define the jetting behavior, which can be described by the Weber number (*We;* Figure 1Ci) [19]. This dimensionless number represents the ratio of inertial to surface tension forces, as described in Equation (1), where ρ is the fluid density, *v* its velocity, *L* is its characteristic length scale, and σ the surface tension [20, 21].

(1) $$We=\frac{\rho v^{2}L}{\sigma }$$

Equation (1): Weber Number

Therefore, material properties such as viscosity and surface tension play a central role in jetting dynamics and form the basis for subsequent optimization of printing parameters in this study. For a given ink, the *We* number can also be tuned through varying laser energy and ink film thickness (e.g., loading volume) to achieve optimal transfer with clean jetting and Rayleigh breakup into single droplets. The Rayleigh‐Plateau instability, which refers to the mechanisms leading to the breakup of a continuous jet into discrete droplets, is an additional critical factor in jet stability within the optimal *We* range [22, 23]. The distance donor‐receiver (DDR; Figure 1Cii) must therefore be adjusted to ensure reliable transfer of materials [24]. Precise tuning of ink properties, laser energy, ink volume, and DDR is essential to define a reproducible LIFT‐bioprinting window that maintains deterministic, high‐resolution patterning, as will be demonstrated in the subsequent sections.

Time‐resolved high‐speed microscopy of an optimal jet shot at 5 µJ revealed that the LIFT‐bioprinting sequence unfolds within microseconds, visualized by stable liquid jetting and well‐defined droplet breakup (Figure 1Di; Video S1). Streak plot analysis captured the key events of the printing process (Figure 1Dii). Specifically, laser irradiation induces the nucleation of a cavitation bubble, which rapidly expands and grows into a thin first jet. The bubble collapse leads to the formation of a secondary jet that is both slower and wider than the first, ultimately breaking up into a train of discrete droplets with disparate velocities. The resulting prints form planar monolayers of micropatterned droplets.

### 2.2 Patterning Capabilities

To demonstrate that LIFT can be used to implement the rapid transfer of matter with a high degree of design freedom, we printed a video game‐inspired design, as well as the logos of the authors’ institutions (Figure 1E). The CAD‐based prints retained high geometric fidelity (> 95%) relative to the target designs (Figure 1F), underscoring the technique's suitability for applications requiring spatially‐controlled and accurate deposition of biological materials. In particular, the ability to deposit discrete droplets is highly valuable for the fabrication of deterministic microvascular networks.

### 2.3 Establishment of a Quantitative Framework to Evaluate Printability

**FIGURE 2:**
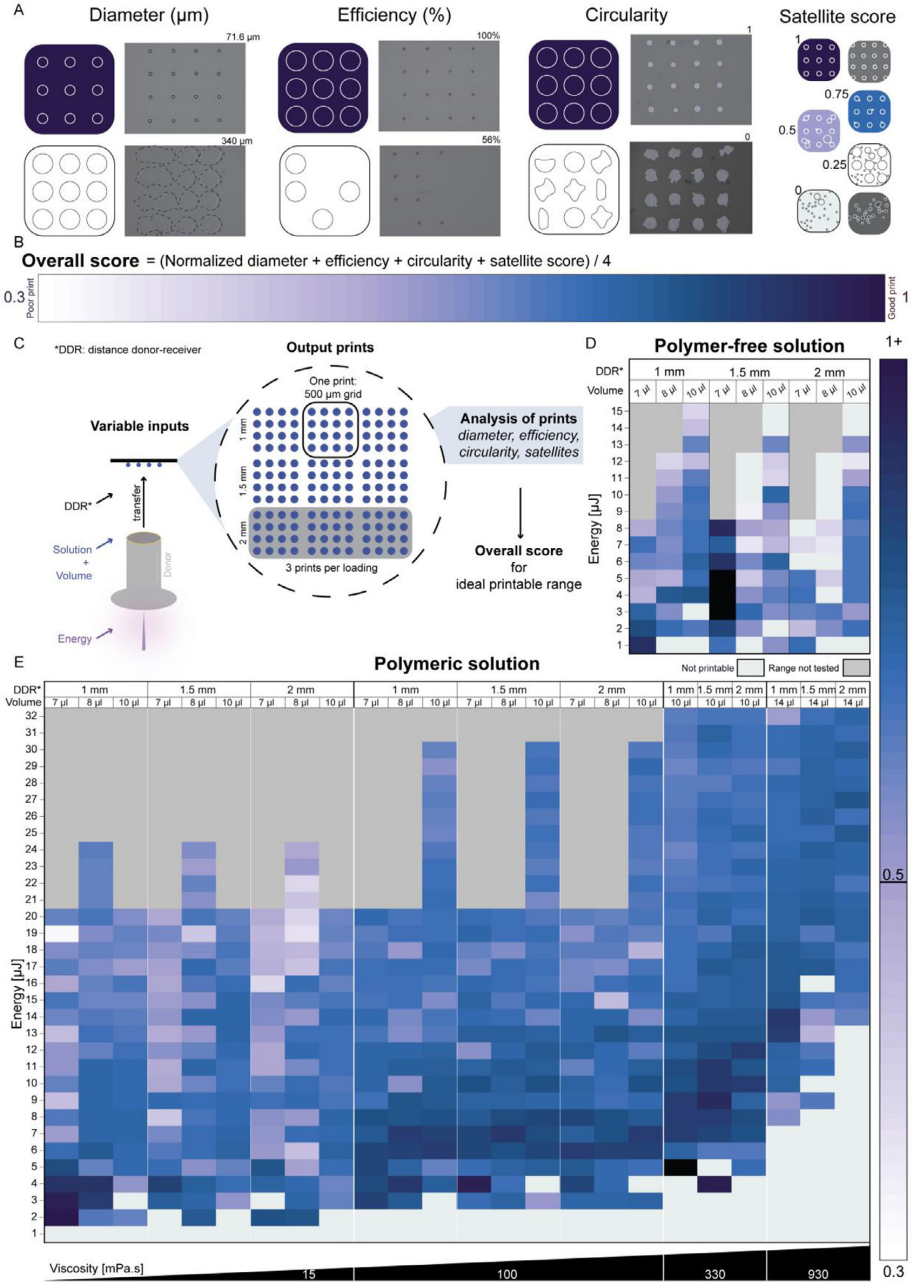
Influence of jetting dynamics on transferred pixel quality. (A) Printed micropatterns were evaluated based on four criteria: droplet diameter, printing efficiency (e.g., completeness of the print), circularity, and satellite score (extent of satellite deposition). (B) An overall score was computed for each condition by normalizing all four criteria over 1 and averaging them. Droplet diameter was inversely normalized relative to the 10^th^ percentile of all diameters obtained from a polymer‐free ink deposited onto a plastic substrate, such that smaller diameters yielded higher scores. (C) Workflow for scoring across an extensive printing matrix. For each solution, laser energy, ink film volume, and printing distance (DDR) were varied. Each individual print consisted of an individual 4 by 4 grid (500 µm spacing). A single pattern is associated with a given solution, energy, and thin‐film volume combination, with each row printed per ink loading, and representing a unique DDR. Therefore, one set of parameters is scored on the basis of 3 prints of a total n = 48 droplets. (D) Overall score map for a polymer‐free solution and (E) overall score map for polymeric solutions with viscosities spanning from 15 to 930 mPa.s. Conditions that were screened but yielded no transfer on the receiver are shown in light gray, unscreened conditions are shown in dark gray. All prints were performed on standard non‐treated plastic tissue culture plates.

While LIFT enables precise transfer of pixelated patterns with design flexibility, it remains essential to identify the parameters that ensure robust printing of complex geometries. To define the optimal printing window of our system, we developed a quantitative framework for assessing printability. We printed an identical pattern across a systematically analyzed set of conditions. We quantitatively evaluated the quality of the prints based on four criteria: droplet diameter, transfer efficiency, droplet circularity, and satellite formation (Figure 2A). Satellites designate the small secondary droplets deposited alongside the main droplet, typically arising from chaotic jetting or complete splashing of the jet onto the receiver surface. These criteria range on a scale from 0 (poor) to 1 (ideal) and were computed into an “overall score”, allowing us to objectively compare print quality across varied experimental parameters (Figure 2B). A score above 0.5 was considered acceptable, as it indicated that each of the four criteria performed above their respective mean value across all tested conditions. This threshold reflects a balanced, above‐average print quality across multiple independent parameters. Equal weighting was applied across the four metrics to ensure that no single parameter dominated the overall assessment. Only the droplet diameter required normalization, for which the smallest measured value was used as a reference, emphasizing relative rather than absolute size. This approach enables fair, reproducible comparisons across conditions and substrates, facilitating generalization of the framework. While some previous studies using LIFT have quantified print outcomes, these were sometimes qualitative and often focused on single‐parameter optimization [24]. In contrast, our scoring approach enables a systematic, multi‐criteria evaluation of print quality across a broad range of experimental conditions. Specifically, we conducted a comprehensive parameter screening, varying ink properties, laser energy, DDR, and volume (Figure 2C). This enabled us to define a printability window as a function of controllable parameters. Droplet transfer was achieved for aqueous (Figure 2D) and polymer‐based inks (Figure 2E). Moreover, printing was achieved across a wide range of viscosities (10 to 10^3^ mPa.s), supporting the broader applicability of this framework for diverse ink systems. Together, these results provide a quantitative basis for predicting optimal transfer regimes across ink formulations.

### 2.4 Printability Window of Low‐Viscous Aqueous Inks

To determine the printable window, we first investigated LIFT transfer using low‐viscous aqueous solutions (polymer‐free ink). Specifically, higher loading volumes (≥10 µL) consistently resulted in higher printability scores, driven by improved transfer efficiency and reduced satellite formation, for example, at 6 µJ (Figure S1). Increasing the loading volumes led to larger droplet diameters, which in turn resulted in higher quality droplet deposition. This was likely due to more homogeneous thin‐film spreading on the donor slide, leading to more uniform transfers, thereby improving print quality. Volume tuning emerged as a critical factor for improving print consistency and quality in aqueous systems.

Droplet deposition of aqueous inks was also strongly influenced by the printing distance: lower DDR values yielded broader printable energy ranges with overall better efficiency and fewer satellites. At 1 mm DDR and 10 µL loading volume, prints produced with energy values between 4 and 11 µJ consistently scored above 0.7, with over 90% transfer efficiency and little to no satellite formation. In contrast, at 1.5 mm DDR and a loading volume of 10 µL, print quality declined with lower transfer efficiency and increased satellite formation, which was exacerbated as laser energy increased. However, the overall printability score remained above 0.5 up to 10 µJ. At 2 mm DDR and the same 10 µL loading volume, droplets became larger, and satellite formation scored slightly lower than at 1 mm, yet printability scores remained above 0.6 up to 10 µJ. Notably, droplet diameters exceeded 200 µm from 8 µJ onward at 1 mm DDR. Overall, shorter DDR values (e.g., 1 mm) yielded better printing accuracy and consistency, likely due to limited jet instability during droplet flight. These results confirm that energy and distance must be co‐optimized to ensure jetting stability and droplet fidelity.

Finally, an optimal energy range between 4 and 10 µJ was defined for polymer‐free inks. Energies above ∼10–11 µJ led to chaotic jetting, increased satellite generation, and inconsistent transfer, while energies below 3–4 µJ resulted in poor transfer efficiency. This suggests that operating within this controlled energy window is critical to ensure stable and accurate printing performance. For low‐viscous aqueous solutions, optimal printability was therefore achieved at higher volumes, lower printing distances, and moderate energies. More generally, lower energy conditions are preferable in bioprinting contexts, as they help preserve cell viability by reducing potentially harmful mechanical effects, such as jet‐induced shear stress, cavitation shockwaves, and impact on the receiver surface [25, 26].

### 2.5 The Effect of Viscosity on the Printability Window

To gain a deeper understanding of the effect of viscosity, we investigated polymer‐based inks with increasing viscosity (Figure S4). These inks enabled exploration of a broader and more complex printability profile, providing insights into how viscous damping influences droplet formation, transfer efficiency, and jet stability [19].

Compared to polymer‐free inks, polymeric solutions exhibited a markedly broader printability window, with print behavior strongly influenced by viscosity. While the precedent statements on increased loading volumes, reduced printing distances, and moderate energies still held true, increasing viscosity notably expanded the printable energy range. For example, overall scores above 0.7 were observed at energies up to 32 µJ for inks with viscosities of 330 and 930 mPa.s.

Satellite formation was also largely reduced with increasing viscosity. Inks with viscosities ≥ 330mPa.s consistently yielded satellite scores above 0.5 (Figure S2), indicating improved jet stability and reduced satellite droplet formation. Even at 100 mPa.s, most conditions scored above 0.5, although a few marginal scores (0.4–0.5) were observed. The broader energy tolerance of polymeric inks likely results from viscous damping, which stabilizes the jet and therefore improves droplet control [27]. Increased viscosity also enabled the printing of smaller‐diameter droplets, attributed to both reduced spreading on the receiver surface and lower volume transfer driven by viscous drag, compared to aqueous inks at equal energy and DDR.

Circularity remained high across all conditions but was particularly improved at lower energies, probably due to increased jet stability and reduced impact velocity (Figure S2c). However, higher viscosity was also correlated with an increased minimal energy threshold required to initiate material transfer. In practice, film homogeneity on the donor slide became harder to maintain at low volumes, requiring minimum loading volumes of ≥14 µL for high‐viscosity inks to ensure uniform film coverage. Despite this, at 930 mPa.s, transfer efficiencies improved considerably from 6 µJ onward and remained consistently high at elevated energies, confirming the robustness of high‐viscosity formulations when sufficient energy and proper film formation are ensured.

Repeatability across all print conditions was generally high, as reflected by the coefficient of variation in droplet diameter (Figure S3). While most conditions showed consistent performance, punctual high standard deviations were observed. These may be attributed to human or system error, such as uneven ink spreading on the donor slide or laser instability. These findings further support the robustness of the scoring method across diverse ink types and parameter ranges.

We believe this comprehensive overview enables the reproducible assessment of transfer quality, an essential requirement for bioprinting applications, where pattern fidelity, transfer efficiency, and minimal mechanical perturbation are critical to preserving cell integrity. This depth of characterization must now be extended to include droplet content to support controlled biofabrication of in vitro constructs.

### 2.6 Predictive Control of Single and Multi‐Cell Droplet Content

**FIGURE 3:**
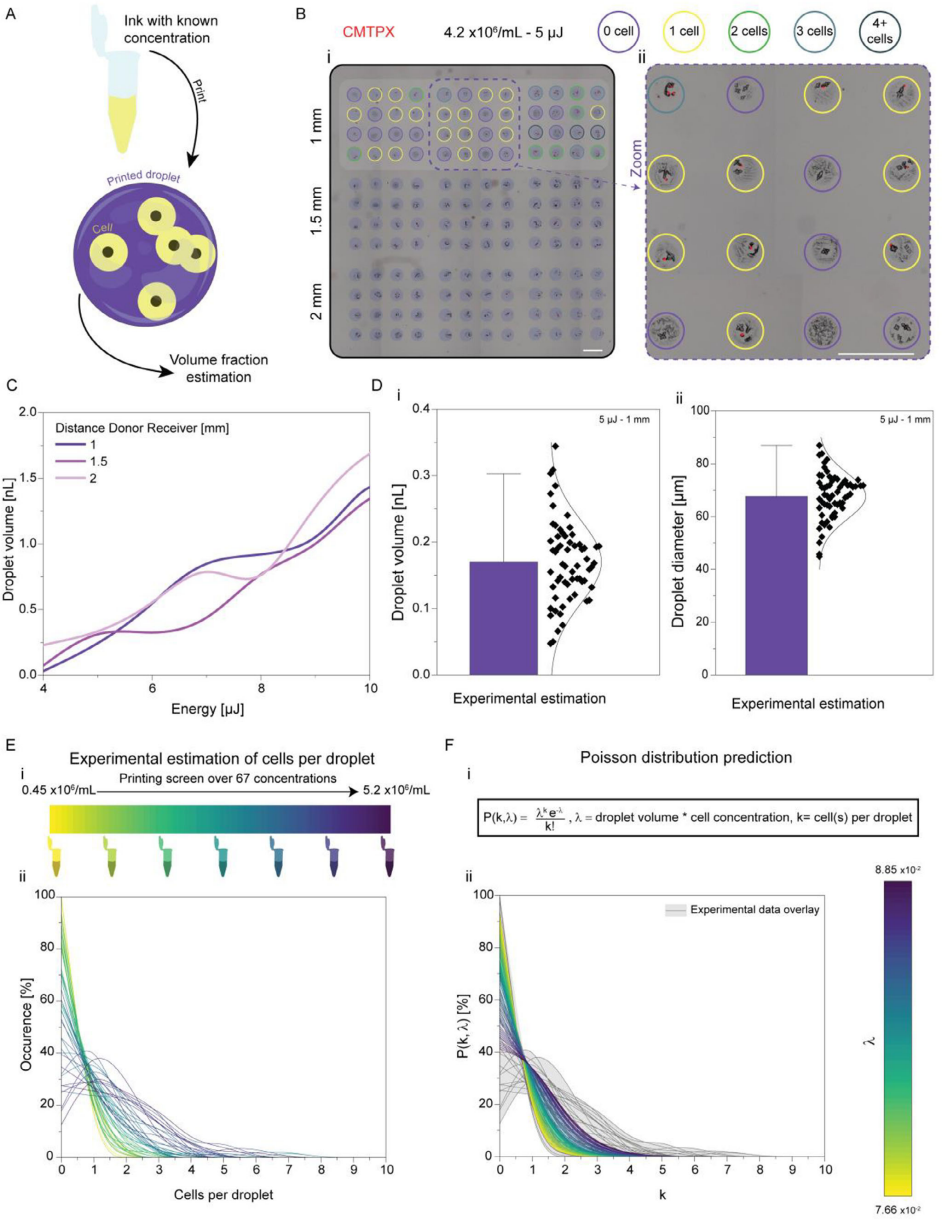
Control over transferred pixel content down to the single‐cell level. (A) Experimental estimation of droplet volume using inks of known cell concentration. After quantifying cell counts per droplet, the droplet volume was calculated back as a volume fraction of the printed ink. (B) (i) Composite fluorescence/brightfield image illustrating the approach applied to a 4.23 × 10⁶ cells/mL suspension of CMTPX‐labeled 3T3 fibroblasts, printed at 5 µJ across varying DDR. (ii) Zoomed‐in region. (C) Mapping of droplet volume as a function of energy and printing distance, analysis based on 3 prints per condition, consisting of total n = 48 droplets. (D) Closer examination of droplet properties at 5 µJ, 1 mm DDR: (i) experimental estimations of droplet volume and (ii) corresponding droplet diameter. Both plots depict the distribution of individual droplet measurements. Analysis based on n = 62 concentrations of each 3 prints and a total 48 droplets per condition (E) Screening of droplet content at the same printing condition across 3T3 fibroblasts containing bioinks at 0.45 to 5.2 × 10^6^ cells/mL. (i) Experimental design and (ii) resulting cell per droplet occurrence for each solution. Analysis based on 3 prints per condition consisting of total n = 48 droplets. (F) Poisson distribution prediction of cell containing droplets: (i) equation, where droplet volume and cell concentration are experimental inputs, and (ii) predicted distributions using the experimentally determined volume (0.17 nL) across the tested concentration range, at 5 µJ, 1 mm DDR. All scalebars indicate 500 µm. All prints were performed on standard non‐treated plastic tissue culture plates.

To investigate the control over droplet content and cell deposition under optimized LIFT conditions, we estimated the volume of individual droplets based on cell counts and known ink concentrations (fixated and CMTPX‐labeled 3T3‐fibroblasts suspensions printed on standard non‐treated plastic tissue culture plates; Figure 3A,B). This approach allowed us to quantify how energy affected the transferred volume of an aqueous suspension across varying printing distances (Figure 3C). Within the 4 to 10 µJ range, we observed a tenfold increase in volume from hundreds of picoliters to a few nanoliters, independent of DDR. To characterize conditions suitable for cell printing, we focused on 10 µL volume, 5 µJ energy, and 1 mm DDR: a low‐energy and short distance regime selected to minimize mechanical stress while maximizing positional accuracy. Under these conditions, the average droplet volume was ∼170 pL, corresponding to a diameter of ∼70 µm (Figure 3D). These values were estimated assuming spherical geometry and are independent of surface hydrophobicity, as they do not rely on contact area or spreading. This robust approach supports the generalization of printing parameters across different receiver types.

To assess the distribution of cells within droplets, we experimentally screened a range of ink concentrations and counted the number of cells per droplet (Figure 3E). The observed distribution closely matched Poisson predictions computed based on the previously estimated droplet volume. These findings confirm the probabilistic nature of cell loading and the predictable behavior of the system at defined droplet volumes (Figure 3F), consistent with prior observations in microfluidic and LIFT systems [28, 29]. Although this analysis was based on a specific printing condition, the strong alignment with the Poisson distribution, together with our extended droplet volume characterization, supports extrapolation of droplet loading outcomes across other energy and concentration combinations.

Single‐cell positioning is valuable to a number of high‐precision cellular applications, such as clonal expansion arrays, single‐cell biochemical screening, and drug‐testing cellular arrays [30, 31, 32]. In biofabricated constructs, the ability to isolate single‐cells from subpopulations is particularly relevant for investigating specific tissue and disease models, such as cancer initiation [33, 34, 35]. Conversely, other applications require controlled high‐concentration cell deposition to promote cell‐cell contact and tissue maturation [36, 37]. To address both needs, we expanded our screening to a broader range of cell densities (Figure S6). Results showed that the transferred volume was impacted by ink concentrations. Based on observed distributions, we defined a low concentration regime as inks ≤10^7^ cells/mL and a high concentration regime as >10^7^ cells/mL, corresponding respectively to conditions favoring single‐cell versus multicell droplet content. Together, these findings establish a flexible framework adaptable to both single‐cell precision and high‐density tissue engineering applications. In the context of vascular patterning, low cell concentrations limit sprouting and interconnections, whereas higher concentrations promote these processes. Consequently, per‐droplet cell loading is critical, and we therefore favoured higher cell densities in the following experiments.

### 2.7 LIFT Bioprinting Allows for High Cell Viability

We next optimized cell printing parameters for high‐density deposition based on insights from previous sections. Parameter choices were informed by our printability mapping and further tuned through adjustments in laser energy and application volume. Additionally, we investigated the effect of receiver material composition on post‐printing cell viability.

**FIGURE 4:**
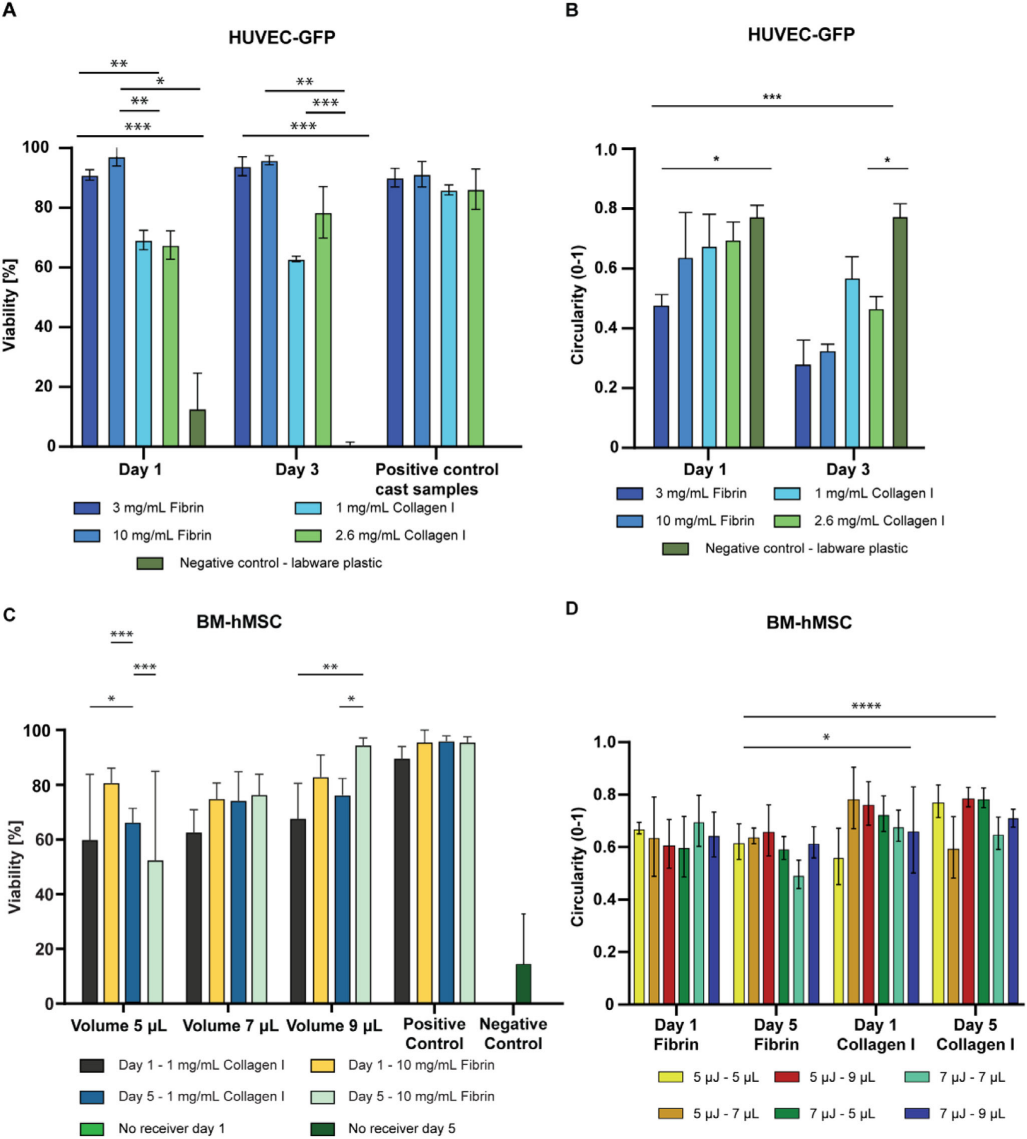
Cell viability and morphology post LIFT printing. (A) Viability quantification of HUVEC‐GFP. Cells were suspended in complete EGM‐2 medium at 30 × 10⁶ cells/mL and LIFT‐printed on five different receiver material compositions: 3 and 10 mg/mL fibrin, 1 and 2.6 mg/mL collagen type I, and uncoated plastic labware (negative control). Positive controls were produced by pipetting 2 µL of cell suspension on hydrogel‐coated receivers. Printing parameters: laser energy 7 µJ, DDR 1.5 mm, application volume 7 µL. Quantification performed on Live Dead staining via Fiji (ImageJ 1.8.0.) for particle analysis, n=3. (B) Cell morphology of HUVEC‐GFP post‐LIFT. Circularity quantifications over the viability dataset of A, n=4. (C) Viability quantification of BM‐hMSC. Cells were suspended in complete MSC expansion medium at 30 × 10⁶ cells/mL and LIFT‐printed at a laser energy of 7 µJ, DDR of 1.5 mm, and application volumes ranging from 5 to 9 µL. Receiver hydrogels were 1 mg/mL collagen type I and 10 mg/mL fibrin, and uncoated labware plastic as a negative control. Controls were prepared as described in A. Quantification performed on Live Dead staining via Fiji (ImageJ 1.8.0.) for particle analysis, n=6 (D) Cell morphology of BM‐hMSC. Circularity quantifications over the viability data set of C, n=4. Samples were LIFT‐printed at DDR 1.5 mm. All statistical analysis were performed via comparisons of the means of more than two populations, two‐way ANOVA in GraphPad (Prism 10.6, USA). Statistical significance is displayed as *p* < 0.05 = ^*^, *p* < 0.01 = ^**^, *p* < 0.001 = ^***^, *p* < 0.0001 = ^****^.

To study the effect of the receiver substrate, HUVEC‐GFP were LIFT‐printed at 7 µJ, 1.5 mm DDR, and 7 µL application volume onto four different hydrogel compositions, followed by viability assessment (Figure 4A). Both fibrin conditions showed significantly higher post‐LIFT cell viabilities than the collagen type I formulations, with 97% viability at both day 1 and day 3 (Figure 4A; complete statistical significance tables in Supporting Information). The cell viability post‐printing on fibrin receivers showed no statistically different results compared to positive controls, while printing onto uncoated plasticware resulted in a drastic viability drop to 5% at day 3. Cells printed directly onto stiff plastic substrates collided with the receiver with a strong mechanical impact while carrying high terminal velocity, compromising viability. In contrast, the presence of a cushioning hydrogel layer helped dampen the impact and preserve cell viability and integrity. The effect of receiver material was further explored by quantifying key morphological parameters (Figure 4B). These revealed lower cell circularity in cells printed onto fibrin compared to collagen, indicating greater spreading, interaction with the substrate material, and stretching to connect to each other (Figure S7). No statistically significant differences were observed between 3 and 10 mg/mL fibrin. Nevertheless, 3 mg/mL fibrin resulted in soft gels that would become unstable early during culture and were highly prone to contraction, whereas the higher fibrin concentration remained stable over longer culture periods. Therefore, 10 mg/mL fibrin was determined to be the optimal receiver material to LIFT‐print HUVEC under these conditions.

For BM‐hMSC, we investigated the compounding effects of laser energy (5 vs. 7 µJ), application volume (5, 7, and 9 µL), and receiver material composition (10 mg/mL fibrin vs. 1 mg/mL collagen) on cell viability. Figure 4C shows the quantification of results obtained at 7 µJ, results for 5 µJ can be found in Figure S7B, and the complete statistical table in the Supporting Information. Across conditions, BM‐hMSC printed on fibrin receivers showed significantly higher cell viabilities than those printed on collagen or uncoated labware plastic. Notably, the 5 µL condition exhibited larger standard deviations, likely due to challenges in applying a homogeneous layer in the donor slide at low application volumes. The addition of BM‐hMSC at high concentrations further increased the viscosity of the carrier medium and compromised the spreading of a homogeneous flat donor layer, impacting transfer efficiency and reproducibility [25]. Increasing the application volume to 9 µL significantly improved viability compared to 5 µL, while the 7 µL condition approached similar levels. Nevertheless, statistical comparisons were limited by the large standard deviations at 5 µL.

Across all conditions, collagen receivers led to lower viability on day 1, followed by recovery by day 5. Furthermore, cells printed on fibrin displayed significantly lower circularity, indicating a more stretched phenotype and suggesting improved interaction with the matrix. Overall, fibrin receivers consistently supported higher cell viability and more favorable morphology. Results obtained for 7 µJ, 7 µL at day 5 remained non‐significantly different from the 7, 9 µL condition, indicating that 7 µL is a suitable application volume for LIFT‐printing while reducing cell waste. Therefore, these results highlight 7 and 7 µL as an optimal set of parameters to LIFT‐print BM‐hMSC on fibrin, with viabilities reaching 97% 5 days post‐printing, equal to positive controls where cells were cast on each indicated hydrogel composition.

### 2.8 Effect of Cellular Density and Inter‐Droplet Spacing on Vascular Network Array Interconnectedness

**FIGURE 5:**
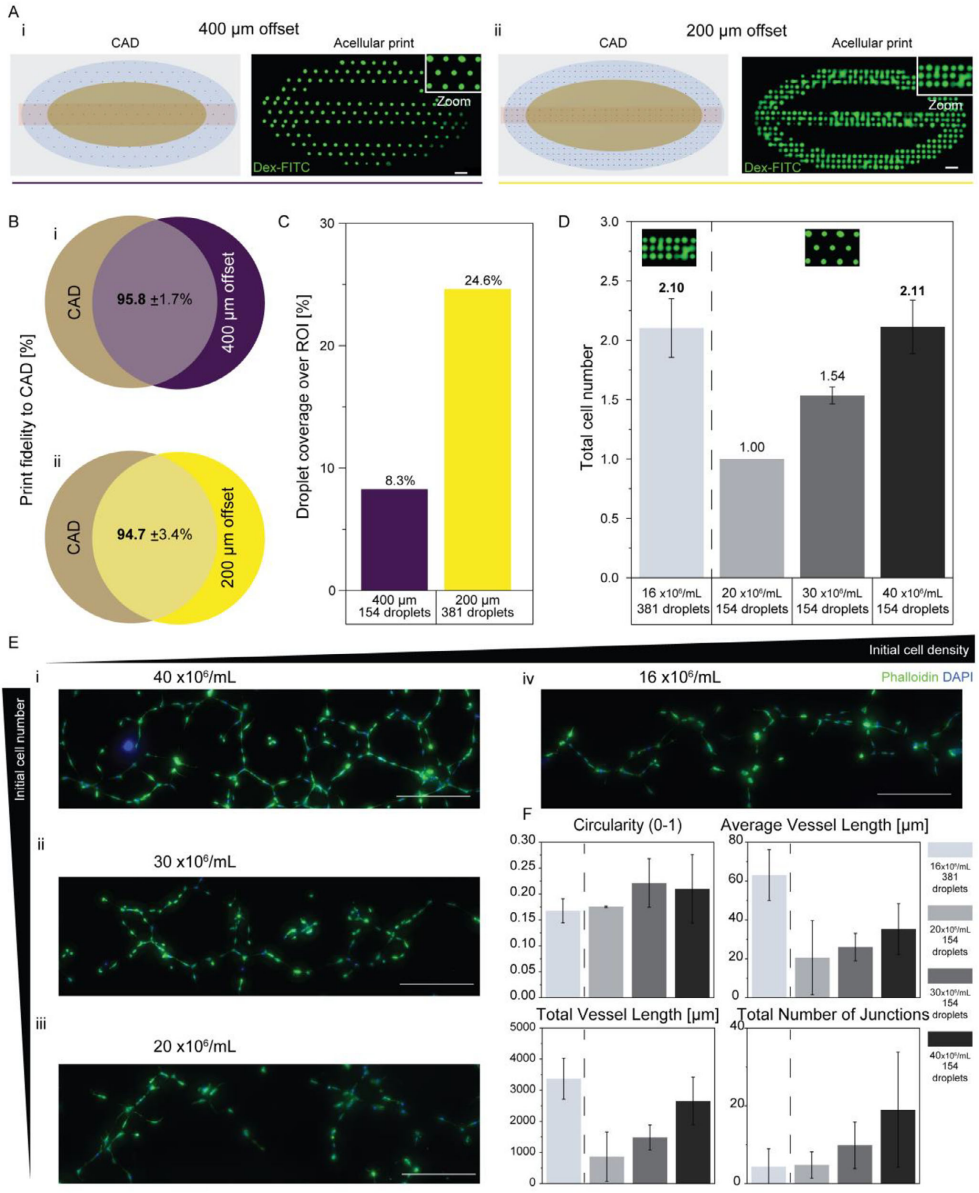
Designing vascular network arrays with controlled cellular density and inter‐droplet spacing. (A) CAD layouts and corresponding acellular prints of Dextran‐FITC illustrate two vascular‐mimetic patterns with droplet spacings of (i) 400 µm and (ii) 200 µm, visualized by fluorescence microscopy. (B) Corresponding print fidelity, quantified as deviations between target and actual inter‐droplet distances for the (i) 400 µm or (ii) 200 µm offset patterns. Analysis based on n = 50 measured inter‐droplet distances. (C) Quantification of droplet coverage area over a shared region of interest (ROI) for both investigated patterns. (D) Relative total number of cells per printed pattern, across different HUVEC‐GFP bioink concentrations. The 200 µm pattern (381 droplets) was printed with a 16 × 10⁶ cells/mL bioink; the 400 µm pattern (154 droplets) was printed with 20, 30, or 40 × 10⁶ cells/mL bioinks. Cell numbers were normalized to the total number of printed cells in the 20 × 10⁶ cells/mL condition (set to 1), enabling comparison across conditions. Analysis based on N=4: estimation of one experimental print per condition and Poisson distribution prediction (3 different models respectively, based on minimum, average, and maximum droplet volume estimated experimentally for high‐concentrations through screening of 29 cell concentrations ranging from 11 to 50 × 10⁶ cells/mL and n=48 droplets per condition). 16 and 20 × 10^6^ cells/mL, 40 and 20 × 10^6^ cells/mL are significantly different at the 0.001 level and at the 0.01 level between 30 × 10^6^ cells/mL and the other conditions (ANOVA one‐way, Tukey). (E) Fluorescence microscopy images of HUVEC‐GFP printed on Matrigel, fixed two days post‐printing, and labeled with DAPI and phalloidin. Zoomed‐in region (i‐iii) 400 µm offset pattern at decreasing cell concentrations. (iv) 200 µm offset pattern at the same cell number as (i), but higher cell density. (F) Quantification of endothelial morphometrics across conditions at day 2 post‐printing. Circularity showed no statistically significant differences (n=2 for the 16, 20, and 40 × 10⁶ cells/mL conditions, n=3 for the 30 × 10⁶ cells/mL condition; 50 to 362 cells considered per sample). Total vessel length (values in micrometer) was significantly greater in the 16 × 10⁶ cells/mL condition compared to both 20 and 30 × 10⁶ cells/mL (p=0.001), and in the 40 × 10⁶ cells/mL condition compared to 20 × 10⁶ cells/mL (p=0.01). Average vessel length (values in micrometer) was also significantly higher in the 16 × 10⁶ cells/mL condition compared to both 20 and 30 × 10⁶ cells/mL (p=0.001 and p=0.01, respectively). The number of junctions was significantly higher in the 40 × 10⁶ cells/mL condition compared to both the 16 and 20 × 10⁶ cells/mL conditions (p=0.05). Vessel lengths and junction analysis based on the different field of views, where n=7 for the 20 × 10⁶ cells/mL (3 prints) condition, n=3 for 30 × 10⁶ (2 prints) and 40 × 10⁶ cells/mL (1 print) conditions, and n=6 for the 16 × 10⁶ condition (1 print). Statistical differences were estimated through ANOVA one‐way Tukey mean comparisons. All scale bars indicate 500 µm.

To evaluate LIFT's ability to pattern endothelial cells with spatial control, we investigated how inter‐droplet spacing and droplet cell concentration influence early vascular network formation. Two array designs were created, with inter‐droplet distances of 400 µm (Figure 5Ai) and 200 µm (Figure 5Aii). Both layouts retained high geometric fidelity to the target designs (>94%, Figure 5Bi‐ii). As expected, the 200 µm offset pattern produced a denser pattern by increasing the number of droplets per region of interest (ROI), resulting in a threefold increase in surface coverage compared to the 400 µm layout (Figure 5C).

We next assessed how varying the total number of cells per pattern impacted network formation. The 400 µm droplet spacing pattern was printed at HUVEC‐GFP suspension concentrations of 20, 30, and 40 × 10^6^ cells/mL (Figure 5D). Additionally, a condition with droplets spaced at 200 µm was included for a 16 × 10^6^ cells/mL ink, which resulted in a similar total cell number as the 40 × 10^6^ cells/mL, 400 µm spacing condition. This allowed us to isolate the effect of pattern density. Cell number estimates were based on Poisson model predictions across a range of droplet volumes (minimum, average, and maximum) under defined print conditions (Figure S6), accounting for the high variability in droplet volume at high concentrations.

To assess how droplet concentration and inter‐droplet spacing influenced endothelial organization, we printed HUVEC‐GFP onto a 1 mm Matrigel sheet, a matrix widely reported in literature to support endothelial sprouting and adopted in vasculogenic assays. Matrigel was thus selected for its established ability to support early vascular morphogenesis, rather than as a comparative receiver material [38, 39]. This choice allowed us to focus on the effect of pattern parameters without the added complexity of varying donor‐ink and receiver compositions, which are optimized later in this study. The printed networks were evaluated two days post‐printing, where cells were imaged and qualitatively assessed for sprouting and interconnectivity (Figure 5E). Cells printed with lower concentrations (20 × 10^6^ cells/mL) appeared rounder and mostly isolated, whereas those at 30 and 40 × 10^6^ cells/mL exhibited pronounced interconnections. Notably, the 16 × 10^6^ cells/mL 200 µm condition demonstrated similar or superior apparent connectivity, suggesting that a denser spatial layout can compensate for lower droplet content.

These observations were further corroborated quantitatively, using morphometric and network‐based metrics (Figure 5F). Cellular shape was evaluated via circularity, with lower values indicating more elongated, branched morphologies. The analysis confirmed that cells were stretching on Matrigel across all conditions (no statistical differences), confirming the suitability of this receiver substrate for the patterning of endothelial cells.

Network integration was assessed via total vessel length, average vessel segment length, and junction number. These metrics, output by AngioTool software, allow us to quantify the lengths of the vessel‐like structures created. Total and average “vessel lengths” showed clear dependence on both droplet concentration and pattern density. Specifically, the 16 × 10^6^ cells/mL condition exhibited significantly greater total and average “vessel lengths” compared to the 20 and 30 × 10^6^ cells/mL conditions (*p* < 0.01). Nevertheless, results were not significantly different from the 40 × 10^6^ cells/mL group. Additionally, the 40 × 10^6^ cells/mL condition showed greater total “vessel length” than the 20 × 10^6^ cells/mL group (*p* < 0.01). Finally, the number of junctions, a proxy for network branching and complexity, was highest in the 40 × 10^6^ cells/mL condition, significantly more than both the 16 and 20 × 10^6^ cells/mL groups (*p* < 0.05). This indicates that increased cell content per droplet enhances local network complexity. For some conditions, quantitative analysis was performed only on single technical replicates. However, additional technical and biological replicates consistently showed the same qualitative trend of increased connectivity at higher cell density. Representative images of these are included in Figure S8.

While the exact position of a single‐cell within the droplet (e.g., at the center or periphery) may vary, maintaining high pattern fidelity ensures that this local variability is minimized. In practice, this means that two droplets containing cells will not necessarily contribute equally to the developing network. To maximize connectivity, each droplet should ideally contain more than one endothelial cell, thereby reducing per‐droplet variability, which we control through Poisson predictions. In parallel, shorter inter‐droplet distances further enhance cell‐cell contact beyond individual droplets. Together, these results show that higher densities enhanced connectivity and morphogenesis, demonstrating that both cell number per droplet and spatial distribution govern early vascular assembly, providing a foundation for designing structured endothelial patterns using LIFT.

### 2.9 Establishment of Vascular‐Like Co‐Cultures via LIFT

We used LIFT to directly co‐pattern HUVEC‐GFP and BM‐hMSC to form multicellular microvascular‐like networks with high spatial complexity. Using previously optimized printing parameters, we assessed whether spatially organized co‐cultures maintain microvascular‐like structural and hierarchical features over several days, and the influence of receiver composition over network stability. While previous studies have reported on LIFT‐printed endothelial cells onto receivers containing BM‐hMSC to provide network support, we demonstrate here that simultaneous, spatially defined patterning of both cell types guides their self‐assembly into hierarchical, branched vessel‐like structures across diverse hydrogel substrates [17].

**FIGURE 6:**
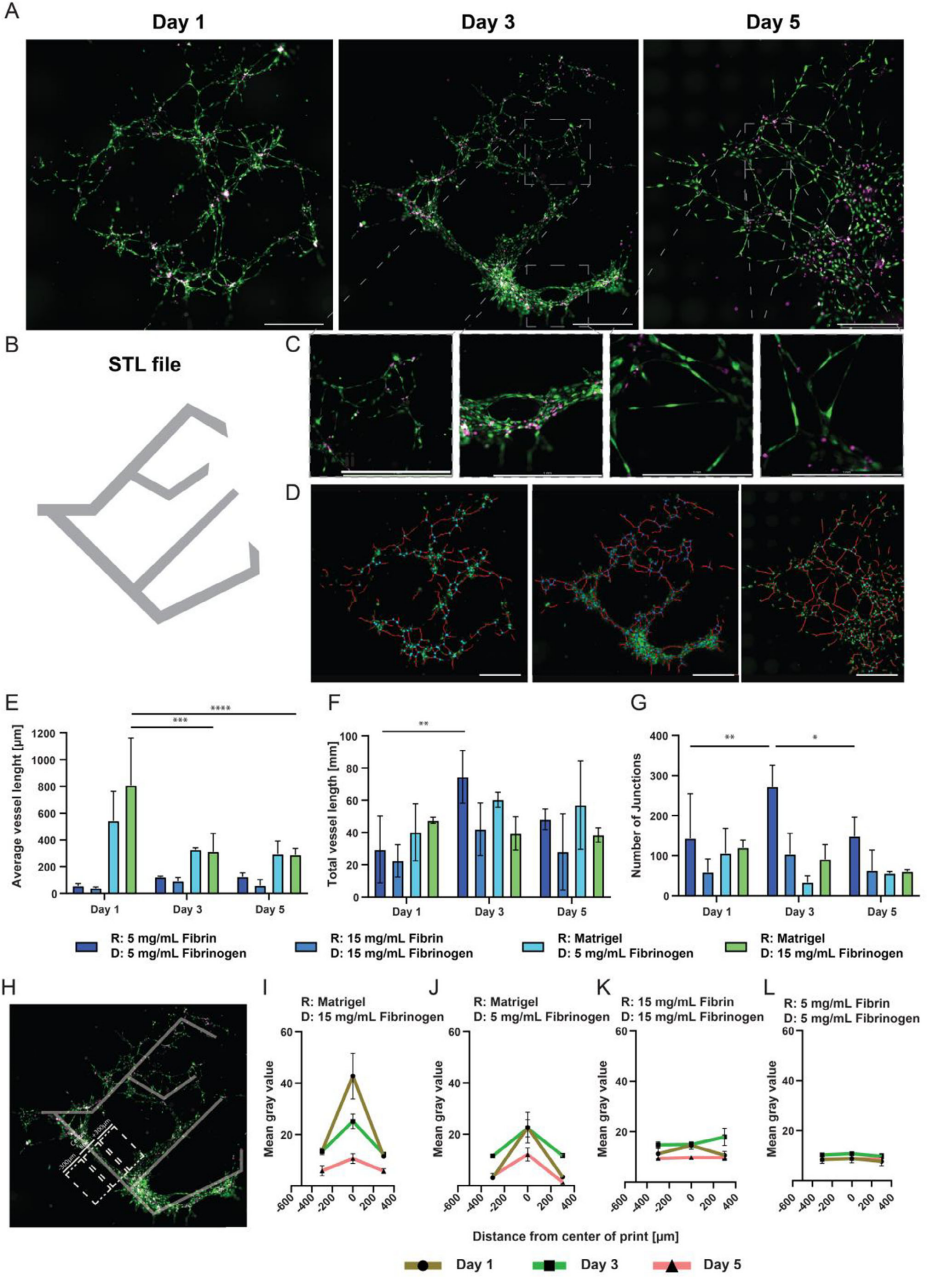
Establishing co‐cultures via LIFT with high spatial complexity. (A) HUVEC‐GFP (green) and BM‐hMSC (magenta) LIFT‐printed on a receiver of Matrigel. All Matrigel receivers are supplemented with 1 U/mL Thrombin. Cells were suspended at a 3:1 ratio HUVEC:BM‐hMSC in 15 mg/mL fibrinogen. Images taken on a Thunder microscope (Leica Microsystems, Germany) at days 1, 3, and 5 post‐LIFT printing. (B) STL file used for LIFT‐printing. (C) Micrographs corresponding to images presented in A. (D) Skeleton overlay produced with AngioTool software to quantify vessel network formation; vessel lengths are shown in red, and cell‐cell junctions in blue. (E–G) Quantification via AngioTool of (E) average vessel, (F) total vessel length, and (G) number of junctions. n=3 for each metric. (H) LIFT‐print image of a Matrigel receiver with cells printed in a 15 mg/mL fibrinogen suspension. Overlay shows STL file mesh and the regions of interest (ROI) selected for quantification of mean gray values as a measure of local cell accumulation. ROI 1 is localized on the center line of the STL overlay (0 µm), ROI 2 to +300 µm, and ROI 3 to ‐ 300 µm from the center line. n=5. (I–L) Mean gray value quantification of LIFT‐prints performed under different receiver (R) and donor compositions (D), analyzed as described in H: (I) Matrigel receiver, 30 x 10^6^ cells/mL suspended in 15 mg/mL fibrinogen. n=5. (J) Matrigel receiver, 30 x 10⁶ cells/mL suspended in 5 mg/mL fibrinogen. n=5. (K) receiver of 15 mg/mL fibrinogen, 30 x 10⁶ cells/mL suspended in 15 mg/mL fibrinogen. n=4. (L) receiver of 5 mg/mL fibrinogen, 30 x 10⁶ cells/mL suspended in 5 mg/mL fibrinogen. n=4. All scale bars = 1 mm. All prints were performed at laser energy = 7 µJ, application volume = 7 µL, and DDR = 1.5 mm, with a total cell concentration of 30 x 10⁶ cells/mL in a 3:1 ratio of HUVEC‐GFP to BM‐hMSC. R indicates receiver, D indicates donor. Statistical analysis in all panels was performed via comparisons of the means of more than two populations via GraphPad (Prism 10.6, USA). Statistical significance test one‐way ANOVA, Tukey multiple comparisons. Significance as *p* < 0.05 = ^*^, *p* < 0.01 = ^**^, *p* < 0.001 = ^***^, *p* < 0.0001 = ^****^.

Co‐cultures were suspended in fibrinogen at two concentrations (5 and 15 mg/mL) and LIFT‐printed on receiver coatings of Matrigel supplemented with thrombin, allowing local fibrin crosslinking upon impact (Figure 6A; Figure S9C,D) [40]. Similarly, the same cell suspensions were LIFT‐printed on receiver coatings composed of 5 and 15 mg/mL fibrin (Figure S9A,B). Positive controls were generated by pipetting 3 µL of cell suspension onto all hydrogel coatings (Figure S9E–G). LIFT‐printed samples on fibrin receivers resulted in poor pattern retention over culture days (Figure S9A,B; Figure 6K,L). Literature search suggested the use of Matrigel receivers loaded with thrombin to act as a crosslinking agent to locally entrap fibrin hydrogels and cells, which we hypothesized would increase pattern retention [40]. All mentioned Matrigel conditions further mentioned contain 1U/mL of Thrombin. Cells printed on Matrigel receivers formed organized networks that remained visible up to day 5 (Figure 6A; Figure S9C,D) and remained similarly located as their original CAD‐defined position (Figure 6B,I,J). Supporting cells closely associated with endothelial structures and appeared to form tight cell‐cell interactions over time (Figure 6C).

Network architecture was analyzed using skeleton overlays, which confirmed preserved design layout and highlighted vascular‐like micro‐network formation, its length, and branching points (Figure 6D–G). Quantification of average “vessel length” showed that 15 mg/mL fibrinogen on Matrigel resulted in significantly longer average vessel segments than 5 mg/mL fibrinogen on Matrigel and significantly different average “vessel length” compared to all other conditions (Figure 6E; full statistical table in Supporting Information). The total “vessel length” was highest in the 15 mg/mL fibrin condition at day 3, with no significant differences between days 3 and 5, indicating that the microvascular‐like network did not regress by day 5 and was maintained over time. The number of junctions (Figure 6G) increased significantly for the 5 mg/mL fibrin condition over time, peaking at day 3. Trends seen in Matrigel samples remained more stable, with no significant differences in total “vessel length” and number of junctions across days. Therefore, Matrigel samples exhibited a decrease in average “vessel length” while maintaining total “vessel length”, suggesting the formation of an increased number of microvessel‐like structures. When compared to cast controls (Figure S9E–G), samples cast on fibrin receivers colonized the hydrogel, while those on Matrigel receivers clumped in the center area. Quantification (Figure S9H–J) revealed that both average and total “vessel length” peaked at day 1 and significantly decreased by days 3 and 5 for the 5 mg/mL condition, with a similar trend observed at 15 mg/mL fibrin.

Overall, LIFT‐printed samples showed enhanced network organization and stability, as reflected by the greater “vessel lengths” compared to cast controls. This indicates that LIFT enables precise spatial positioning of cells in 2D patterns that promote network formation and maintenance over time.

Quantification of vessel‐like metrics provided insight into the ability of cells to self‐assemble following LIFT‐printing. We further investigated the effect of receiver material on the stability of LIFT‐printed patterns (printing pattern CAD provided in Figure 6B), assessing the ability of cells to remain at their deposited location while maintaining vessel‐like network organization. The strategy for quantifying pattern fidelity is shown in Figure 6H, where the CAD design mesh was overlaid onto the obtained fluorescent images. Three adjacent sections of 300 µm^2^ were selected: one centered on the print, and two positioned at a 300 µm distance on each side. Fluorescent intensity within each region was measured, where a high contrast in pixel intensity indicated a higher concentration of cells within the area. Samples printed on Matrigel receivers with cells suspended in 15 mg/mL fibrinogen (Figure 6I) presented a high concentration of cells within the printed area and a sparse signal in neighboring regions, suggesting that the cells maintain a differential position. Samples printed on Matrigel with cells suspended in a lower concentration of fibrinogen (Figure 6J) showed marked differences in cell positioning, although less pronounced than at the higher fibrinogen concentration. In contrast, samples printed on fibrin receivers (Figure 6K,L) showed no difference in cell density across the analyzed areas. Comparison with the corresponding images (Figure S9A,B) revealed that cells printed on fibrin receivers displayed higher motility and colonized the surrounding hydrogel, leading to loss of pattern definition. These results highlight the importance of selecting an appropriate receiver hydrogel for applications requiring precise micropatterning of vascular‐like co‐cultures.

We have demonstrated the versatility of LIFT to precisely micropattern hierarchical, branching‐like networks of self‐assembled, microvascular‐like structures, guiding cellular self‐assembly into complex geometries that maintain their pattern over at least five days of culture. Such a spatially hierarchical arrangement would be unattainable without the use of a micropatterning technology such as LIFT.

## 3 Conclusions

We introduce LIFT as a high‐resolution, high‐viability micropatterning technique to fabricate pre‐vascular cell‐laden, multicellular constructs. We systematically characterized the printability window of LIFT by comprehensively varying key parameters influencing droplet formation. These parameters, including laser energy, ink volume, viscosity, and distance donor‐receiver, are intricately interdependent, and their combined effects determine droplet fidelity and transfer quality. To capture this interplay, we developed a novel, objective printability score that integrates key droplet metrics into a single indexed score. This quantitative framework lays the foundation for systematic process optimization and enables the prediction of optimal parameters for a given ink formulation.

We further demonstrated that cell distribution within droplets followed a Poisson distribution, enabling deterministic single‐cell patterning with implications for the creation of microarrays or single‐cell studies. Predictability still held true at higher cell densities, where we also achieved high cell viability across multiple cell types and highlighted the importance of both the presence and composition of the receiver material.

Using this foundation, we explored early micro‐vascular morphogenesis as a function of both droplet cell concentration and pattern density. Both parameters were found to enhance endothelial cell sprouting and network interconnectivity. Finally, we applied LIFT to establish co‐cultures of endothelial and supporting cells with high spatial control. By tuning the composition of donor and receiver materials, we preserved printed pattern integrity and sustained microvascular‐like network formation over five days of culture.

Altogether, this work establishes a robust and versatile approach for high‐resolution bioprinting of vascular cell‐laden structures. The proposed method can be readily adapted to various hydrogel systems and incorporate microvascular features into organized tissues for applications in tissue engineering and organ‐on‐chip development. By tuning design parameters, such as donor‐receiver composition, droplet concentration, and pattern layout, we show that early morphogenetic processes can be modulated with precision, offering a platform not only for fabrication but also for the study of vascular development in defined microenvironments. While the present work focuses on early, 2D vessel‐like network formation, as opposed to lumenized 3D vasculature, the same framework provides a foundation for future studies to further explore long‐term network stability, functional integration or perfusion potential in more complex multicellular systems.

## 4 Experimental Section

All % are given as weight/weight unless otherwise specified.

Reagents were purchased from Sigma–Aldrich, Germany, unless otherwise stated.

### 4.1 Laser Printing Setup and Parameter Optimization Rationale

LIFT‐printing was performed using a Next generation bioprinter 4D Research (NGB‐R; Poeitis, France). The core platform consists of a pulsed 1064 nm Nd:YAG laser source, where the beam is shaped and directed via a galvanometric scanner to user‐defined *xy* coordinates, enabling precise spatial control. The laser is focused onto a donor substrate coated with a thin Ti/Au absorbing layer (Poietis, France) and onto which a thin film of ink is pipetted. Upon irradiation, the metallic bilayer is heated and vaporized, effectively converting thermal energy into kinetic energy by generating a high‐pressure cavitation bubble that propels a jet of ink toward a receiver substrate. Three transfer regimes can be identified: a subthreshold regime at low *We*, where no transfer of material occurs because of insufficient energy; an optimal printing zone; and, an above‐threshold region at high *We* that leads to a progressively more chaotic jetting profile, causing splashing of the ink and subsequent loss of accuracy and resolution in the droplet deposition [19, 24, 41, 42]. Printing optimization was therefore completed to situate the writing within the optimal window. Additionally, for a reliable transfer of material, the breakup length of a jet should precede contact with the receiver, for example, be shorter than the DDR. However, when the DDR is too large, it will result in a partial and less accurate transfer. The effect of these distances on print quality where thus included in our study.

The designs were either imported as. stl files into the printer's software (Zebr4D; Poietis, France) or directly designed into the software. The shapes were then filled with a droplet grid, set at a defined inter‐droplet distance. The bio obtained file was then opened on NGB‐R software (Poietis, France), breaking down the single pattern printing into a sequence of multiple jobs to follow (e.g., printing in one or multiple steps with specific laser energy, DDR, irradiation offset on the donor as well as receiver plate's coordinates, and defined breaks).

### 4.2 High‐Speed Imaging Setup

To capture the formation of the cavitation bubble as well as the dynamics of the induced jets, a high‐speed camera (Photron, SA‐XII) was used at a frame rate of 216 kfps with a shutter speed of 1/800000 sec, that is, an exposure time of 1.25 µs. This frame rate and exposure time were used to obtain images sharp enough to detect the very fast dynamics of the events. The maximum camera resolution was used with this frame rate, 640px x 40px. The optical setup allowed a resolution of 3.33 µm/px. The typical duration of the experiments was 1 ms, from bubble formation until the last droplets of the second jet left the frame.

The camera was placed outside the laser printing device, in order to use the device window as a protective filter (1064 nm) for the camera sensor and block the very intense light of the pulsed laser. The illumination was performed in transmitted mode, using a light source (Schott ColdVision CV‐LS) coupled to an optical fiber. The light was placed directly on the sample, and a microscope objective (5x) was used to collect the light and create the image at the camera sensor. The lamp was activated for 2 ms, to avoid changes in the temperature of the liquid on the surface of the donor, which would modify the dynamics of the bubble and the induced jet. The synchronization between the laser, camera, and illumination was done through a modification in the original Poietis software.

A custom Matlab script was used to generate streak images and facilitate data capture.

### 4.3 Identification of a Printability Window and Establishment of an Objective Scoring Framework

A screening for the optimal printing parameters was performed by iterating the laser energy, the distance donor‐receiver, the volume applied on the donor slide, and the viscosity of the ink. In brief, the donor slides are 15 mm diameter optic grade glass double‐coated with titanium and gold, onto which 7 to 14 µL volume was applied with a micropipette. An aqueous solution (0.1% Bovine Serum Albumin, BSA, in Phosphate Buffered Saline, PBS) and polymer solutions of increased viscosity (0.%5 to 2% alginic sodium salt 80–120 cP, Wako, dissolved in 0.1% BSA in PBS) were used. The receiver substrate was regular laboratory‐grade plasticware (Greiner, Germany).

The design was a 3x3 grid of individual 4x4 droplet grids, where each droplet is separated by 500 µm, and one line of the 3x3 grid (n = 48 droplets) was printed per unique set of conditions (ink, volume, energy, DDR). The prints were therefore shot from the left, middle, and right side of the donor for example, accounting for possible inhomogeneous spreading of the ink on the donor. The prints were then captured through an inverted microscope (EVOS) with a 4x objective in brightfield mode. The aqueous and polymer pictures were divided into 2 datasets to train 2 distinctive cellpose models, an open‐source segmentation tool using Python. The respective datasets were then analyzed through cellpose, generating a mask for each picture. These masks were then run through Fiji to analyze printing efficiency, individual droplet diameter, and circularity. The obtained results were manually corrected when necessary.

The satellite score was manually graded according to the set criteria: 0 (only satellites), 0.25 (identified droplets with a lot of small satellites), 0.5 (identified droplets with equally sized satellites), 0.75 (identified droplets and some small satellites), 1 (identified droplets without any satellites).

The droplet diameter was normalized over 1 based on the 10^th^ percentile of droplet diameter (in order to exclude outliers) estimated from aqueous solutions.

For each individual set of parameters, the overall score was obtained by averaging the normalized droplet diameter, printing efficiency, circularity, and satellite score.

### 4.4 Droplet Volume Estimation

The same printed design was used to estimate the droplet volume, calculated according to the following equation:


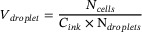

where *N_cells_* is the total number of printed cells, *C_ink_* is the initial ink concentration and *N_droplets_* is the number of droplets analyzed. We based our analysis on the counting of n = 48 droplets per set of conditions (varied energy and DDR). Cells (fixated NIH 3T3) were fluorescently labeled (CMTPX) and identified by inverted microscopy (Zeiss Axio Observer).

### 4.5 Cell Distribution Estimation

Cells (fixated NIH 3T3) were fluorescently labeled (CMTPX), concentrated, and diluted into over 67 solutions of 0.45 × 10^6^ cells/mL to 5.2 × 10^6^ cells/mL. The same grid pattern was printed (5 µJ, 1 mm DDR, 10 µL) with n = 48 droplets per condition (variation of cell concentration) and captured by inverted microscopy (EVOS). The number of cells per droplet in each condition was then assessed and compared to a Poisson distribution model, fit with the obtained droplet volume at the same given printing parameters.

### 4.6 Cell Culture and LIFT‐Printing of Cells

HUVEC‐GFP were purchased from Lonza and expanded to Passage 6–8, EGM‐2 (Lonza, Switzerland) cell culture medium was used following manufacturer's instructions, with 10% Fetal Bovine Serum (FBS, Capricorn Scientific, Germany) replacing Gentamycin for 1% Penicillin/Streptomycin (P/S, Gibco, Germany). Cells were thawed and seeded at 3.000 cells/cm^2^ and sub‐cultured to 85%–90% confluency before passaging using 0.25% Trypsin‐EDTA (Gibco, Thermo Fischer) for 5 min, inactivating with complete medium. Cells were then counted and either sub‐cultured, frozen, or used for experiments. For the cellular density and inter‐droplet spacing parametric study, GFP‐HUVEC were purchased and expanded to passage 6–7 (Angio‐Proteomie) with EGM‐2 medium (Lonza, Switzerland) according to the manufacturer's instructions. Cells were thawed and seeded onto rat tail collagen I‐coated flasks (0.1 mg/mL, Corning, USA) at 6.000 cells/cm^2^ and sub‐cultured to 85%–90% confluency before passaging using 0.25% Trypsin‐EDTA (Gibco, Thermo Fischer) for 3–5 min, inactivating with complete medium. Cells were then counted and either sub‐cultured, frozen, or used for experiments.

Human bone marrow‐derived mesenchymal stromal cells (MSCs) were isolated from bone marrow aspirates of consenting patients, as previously described [43]. Briefly, human bone marrow aspirates were obtained from the iliac crest of patients who were receiving spondylodesis or hip replacement surgery. Isolation and distribution were performed in accordance with protocols approved by the Biobank Research Ethics Committee (isolation 08–001, distribution protocol 18–739, University Medical Center Utrecht). The protocols used were in line with the principles embodied in the Declaration of Helsinki. MSCs were expanded in α‐Modified Eagle Medium culture medium (α‐MEM, Gibco, Life Technologies) supplemented with 10% FBS, 1% P/S, 1% L‐ascorbic acid‐2‐phosphate (ASAP; Sigma–Aldrich, The Netherlands), and 1 ng.mL^−1^ basic fibroblast growth factor (bFGF; R&D Systems) and used at passage 4–5.

Holder adaptors for 12‐well plates were designed using Fusion360 (Autodesk, USA) and printed using a Selective Laser Ablation (SLA) printer, FormLabs 3B+ (FormLabs, USA) and BioMed Clear resin according to the manufacturer´s instructions. Holders were post‐processed according to the manufacturer´s instructions and used to hold the receiver hydrogel and center cartesian print coordinates of the LIFT‐prints.

Cell viability was tested in 3 mg/mL or 10 mg/mL fibrin or 1 mg/mL or 2.6 mg/mL collagen type 1 (Rat tail, Corning, USA) as receiver hydrogels. Fibrin hydrogels were prepared by dissolving fibrinogen from human plasma (Sigma‐Aldrich, Germany) in PBS and diluting Thrombin from human plasma (Sigma‐Aldrich, Germany) in EGM‐2 to a final concentration of 2 U/mL Thrombin. Collagen type I hydrogels were prepared by neutralizing the collagen to pH 8.5 using HCl and diluting in sterile 10X PBS. Hydrogels were mixed and 50 µL were added to the 12‐well plate inserts. Gels were polymerized at 37 °C, for 30 min for fibrin, and 2 h for collagen type 1.

For cell printing, cells were suspended in varying cell concentrations, 1 – 40 × 10^6^ /mL in the respective cell culture medium. Cells were then loaded onto donor slides at volumes ranging from 5 to 9 µL and printed at 5 and 7 µJ laser energy and a distance donor‐receiver (DDR) of 1.5mm. Cells were left to attach to the receiver hydrogel for 30 min at 37 °C prior to adding cell culture medium. Cell viability was assessed at days 1, 3, and 5 post‐LIFT using ethidium homodimer and Calcein AM staining. Images were taken using a Thunder microscope (Leica Microsystems, Germany). Images were analyzed using Fiji, Image J. Statistical analysis was performed using Prism, GraphPad 10.1.

### 4.7 Cellular Density and Inter‐Droplet Spacing Parametric Study

HUVEC‐GFP were suspended at 44.4 × 10^6^/mL and diluted to 33.3 × 10^6^/mL, 22.2 × 10^6^/mL, 17.8 × 10^6^/mL in cell culture medium. For each of the inks, 1 µL of 0.1% BSA in PBS was pipetted on the donor to facilitate spreading, followed by 9 µL of cell suspension, which immediately spread homogeneously on the donor without additional manipulation, therefore diluting the inks to respectively 40, 30, 20, and 16 × 10^6^/mL. For the 40, 30, 20 × 10^6^/mL conditions, the 400 µm pattern was printed with a job sequence consisting of one ink loading and 2 prints (middle branch and side branches). For the 16 × 10^6^/mL condition, the 200 µm pattern was printed with a job sequence consisting of 2 ink loadings and 8 prints, for example, loading every 4 prints (middle branch and side branches at 400 µm offset then in between the droplets to obtain an overall 200 µm pattern) to avoid the interaction between two successive jets placed too close to each other. The acellular patterns were also printed with dextran‐fluorescein and imaged through an inverted microscope (Zeiss Axio Observer).

The cells were printed onto Matrigel (Corning, USA) was the uncured hydrogel, was cast into 14 mm diameter disk molds of 1 mm thickness (custom‐made molds printed with an SLA printer and biocompatible resin, Formlabs) within glass bottom dishes (Cellvis), then left to cure at 37 °C for one hour prior to printing, where molds were removed. Patterns were imaged through inverted microscopy (Zeiss Axio observer and Evident IX83 widefield). On day 2 post‐printing, the samples were fixated, permeabilized with Triton X‐100 (0.1%), and labeled with phalloidin conjugated to Alexa Fluor 488 (50 U/mL) to visualize F‐actin. Nuclei were counterstained with DAPI (0.01 mg/mL). Samples were visualized through inverted microscopy (Evident IX83 widefield) and analyzed via AngioTool software. For the latter, at least three fields of view per condition were analyzed for morphometrics (see figure caption for details). Fields were selected based on initial print quality on day 0, excluding regions with poor initial deposition or focus that could compromise image‐based quantification. Circularity was analyzed through Fiji (ImageJ) on HUVEC‐GFP pictures at day 2 post‐printing, averaging between biological replicates.

### 4.8 BM‐hMSC and HUVEC‐GFP Co‐Culture

Cell preparation: HUVEC‐GFP and BM‐hMSC were subculture as previously described. For live imaging, BM‐hMSC were stained with DiD cell tracker (Sigma‐Aldrich, Germany) for 20 min at 37°C followed by 3 washes in PBS0‐MQ. Cells were then counted and suspended in a 3 to 1 ratio of HUVEC‐GFP to BM‐hMSC in either 5 or 15 mg/mL fibrinogen at 30x10^6^ cells/mL with 7.68 µM aprotinin (Sigma‐Aldrich).

Receiver substrate preparation:

Matrigel was thawed on ice for 2 h. Once thawed, Thrombin as added for a final concentration of 1 U/mL prior to loading in SLA‐printed holders 600 µm height, 5 mm diameter. Hydrogels were incubated at 37°C for 30 min prior to LIFT‐printing.

Fibrin was prepared at 5 and 15 mg/mL with a 1 U/mL final concentration Thrombin and loaded in SLA‐printed holders 600 µm height, 5 mm diameter. Hydrogels were incubated at 37°C for 30 min prior to LIFT‐printing.

### 4.9 LIFT‐Printing

The printing sequence used was 1 print 5 or 15 mg/mL fibrinogen, followed by 2 prints of cell‐containing bioink prepared as described above. Once LIFT‐printing was performed, well plates were incubated at 37°C for an additional 20 min prior to adding 1.5mL complete EGM‐2 medium. Cell‐containing prints were performed twice with an offset of 150 µm. Printing parameters were 7 µJ laser energy, 7 µL application volume, and 1.5 mm DDR.

Live images were captured at days 1, 3, and 5 at 10 and 20x using a Thunder microscope. Branching networks quantification of total and average vessel length and number of junctions was performed using AngioTool software with n = 3 for each analyzed condition. To analyze printed pattern fidelity, the mesh of the original CAD design was overlaid on the obtained live images using Fiji, ImageJ. Mean gray intensity was measured in an area of 300 µm^2^ selected around the CAD design center line and two adjacent regions n = 5. Data was processed using GraphPad Prism 10.6. Statistical analysis was performed using a 2‐way ANOVA for vascular‐like branch formation.

### 4.10 Statistical Analysis

Statistical analyses were conducted using GraphPad Prism (GraphPad Software, Inc., San Diego, CA, USA) and OriginPro (OriginLab, Massachusetts, USA). Data was processed as appropriate and indicated in each figure caption. All data is presented as mean ± SD unless otherwise stated. Comparisons of the means of more than two populations were performed via one‐way or two‐way analysis of variance (ANOVA) for normally distributed datasets and otherwise specified in figure captions. Statistical significance Statistical significance is displayed as *p* < 0.05 = ^*^, *p* < 0.01 = ^**^, *p* < 0.001 = ^***^, *p* < 0.0001 = ^****^. All sample sizes are indicated in the figure captions or graphs as individual data points.

## Conflicts of Interest

The authors declare no conflict of interest.

## Supporting information




**Supporting File 1**: adhm70517‐sup‐0001‐SuppMat.docx.

**Supporting File 2**: adhm70517‐sup‐0002‐Data.zip.

**Supporting File 3**: adhm70517‐sup‐0003‐VideoS1.mp4.

## Data Availability

The data that support the findings of this study are available from the corresponding author upon reasonable request.
