# Supplementary material for: A Quantitative Printability Framework for Programmable Assembly of Pre‐Vascular Patterns via Laser‐Induced Forward Transfer
Source: Adv Healthc Mater. 2025 Nov 21;15(8):e03665. doi: 10.1002/adhm.202503665 (PMC12927535; doi:10.1002/adhm.202503665)
Supplement: Supplementary file 1 — Supporting File 1: adhm70517‐sup‐0001‐SuppMat.docx. [file ADHM-15-0-s001.docx]

**Supplementary information**

Cécile Bosmans ^1#^, Núria Ginés Rodriguez ^2,3#^, Ulisses Jesús Gutiérrez Hernández ^4ǂ^, David Fernandez Rivas ^4^, Marcel Karperien ^1^, Jos Malda ^2,3,5*^, Liliana Moreira Teixeira ^1*^, Riccardo Levato ^2,3,5*^, and Jeroen Leijten ^1*^

# These authors contributed equally to this work. Author sequence randomly determined.

*Corresponding authors

^1^Department of BioEngineering Technologies, Faculty of Science and Technology, TechMed Centre, University of Twente, Enschede, The Netherlands

^2^Department of Orthopaedics, University Medical Center Utrecht, Utrecht, The Netherlands

^3^Regenerative Medicine Center Utrecht, Utrecht, the Netherlands

^4^Mesoscale Chemical Systems group, University of Twente, Enschede, The Netherlands

^ǂ^Current position: Departamento de Física, Facultad de Ciencias, Universidad Nacional Autónoma de México, Mexico

^5^Department of Clinical Sciences, Faculty of Veterinary Medicine, Utrecht University, Utrecht, The Netherlands

The video corresponding to Figure 1Di and ii is attached as supplementary video 1.

Complete statistical table corresponding to cell viability as well as co-culture study are attached as excel files.

1. **Mapping of printability window**

The non-normalized individual scores are included for both polymer-free and polymeric solutions.

**
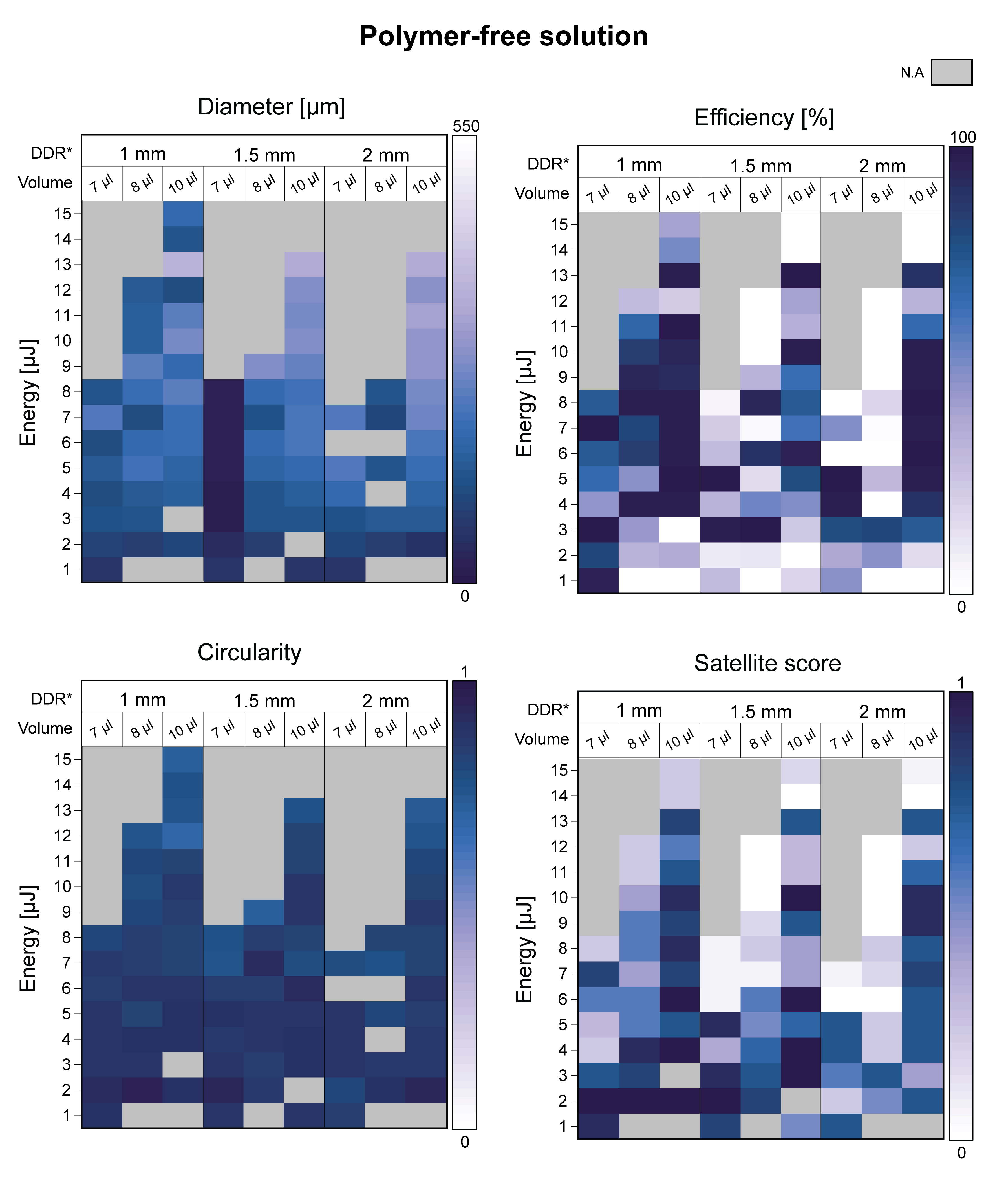
**

Supplementary Figure 1 Individual scores polymer-free solution. DDR: Distance donor-receiver.


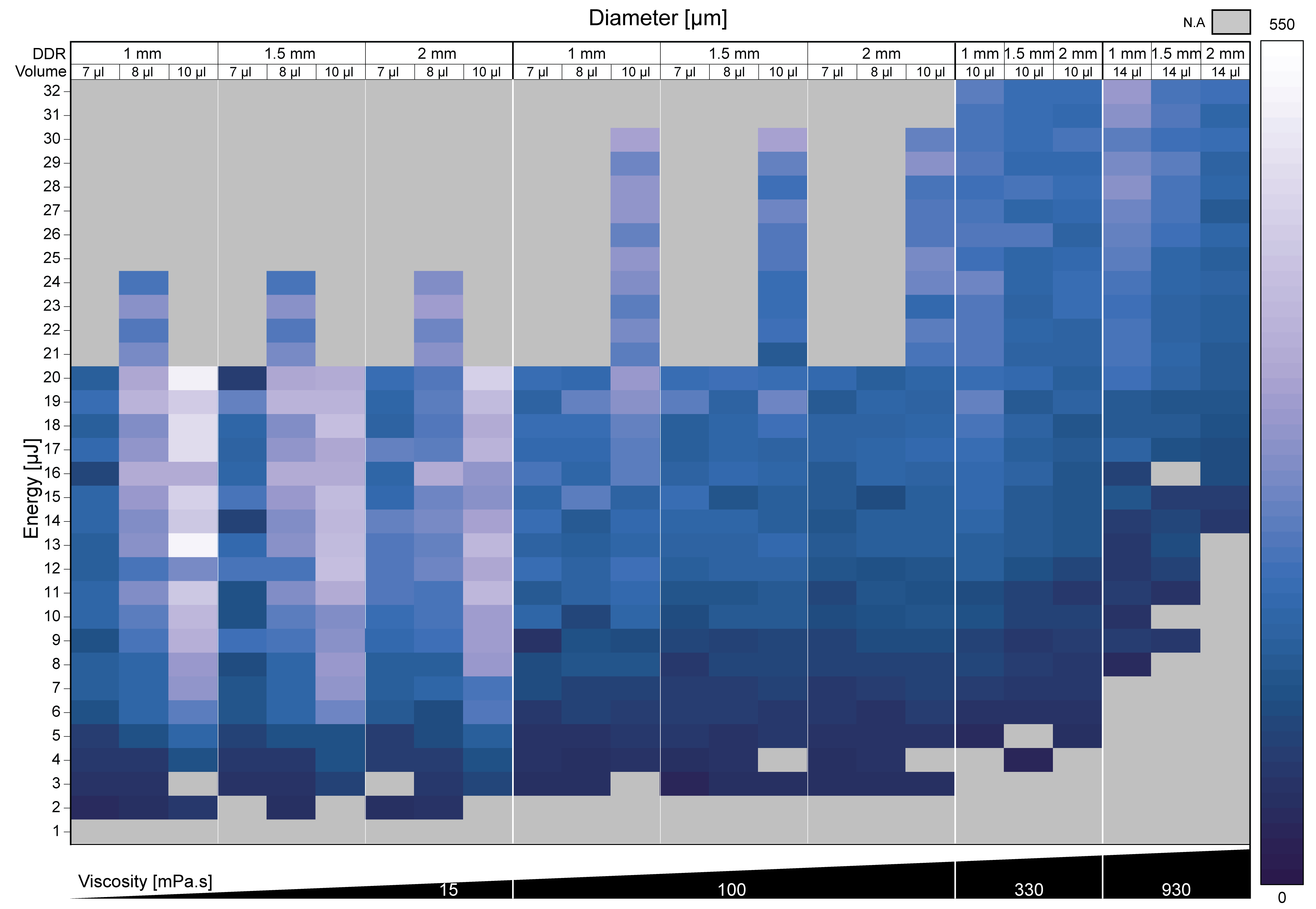


Supplementary Figure 2a Droplet diameter polymeric solution.


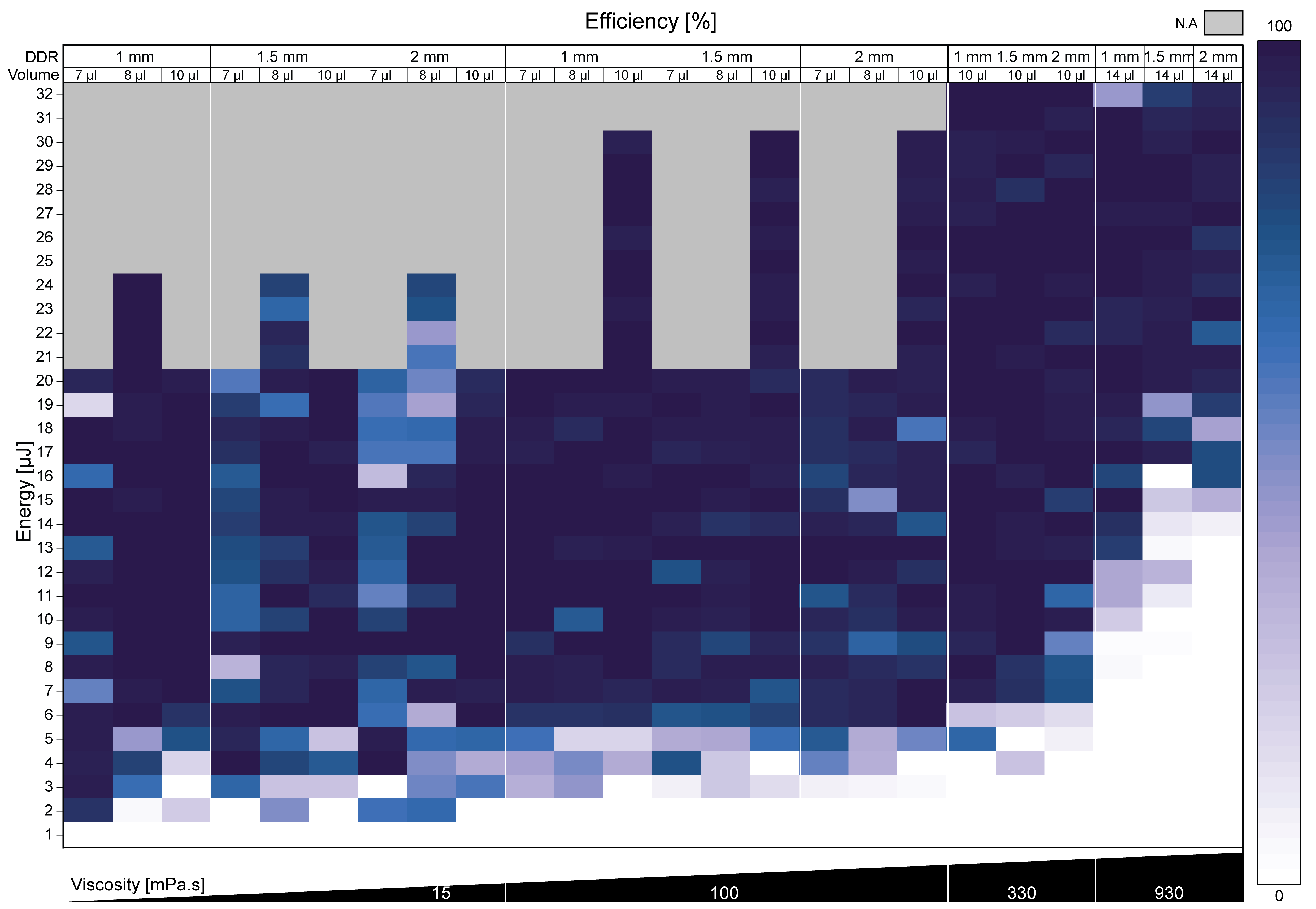


Supplementary Figure 2b Printing efficiency polymeric solution.


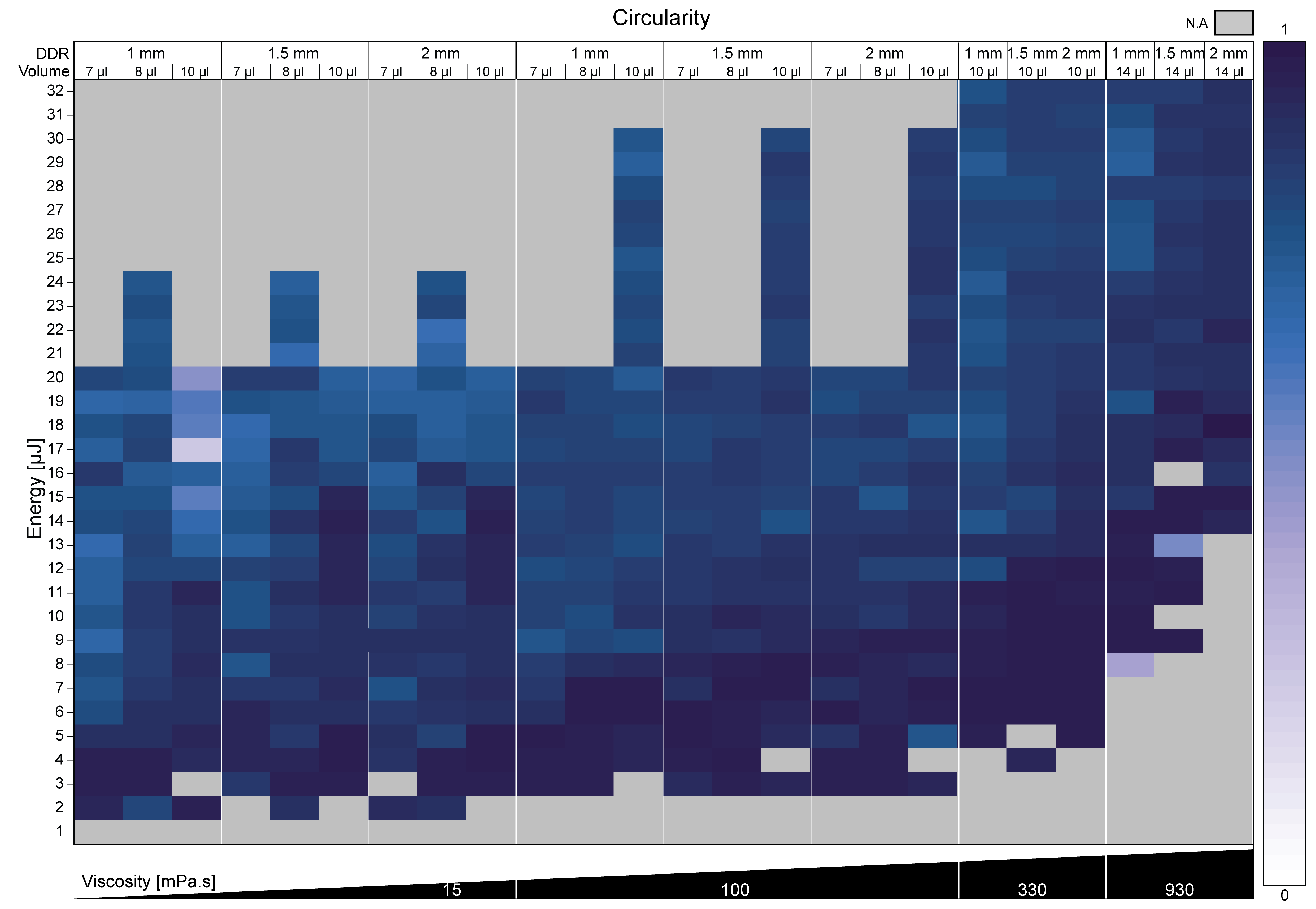


Supplementary Figure 2c Droplet circularity polymeric solution.


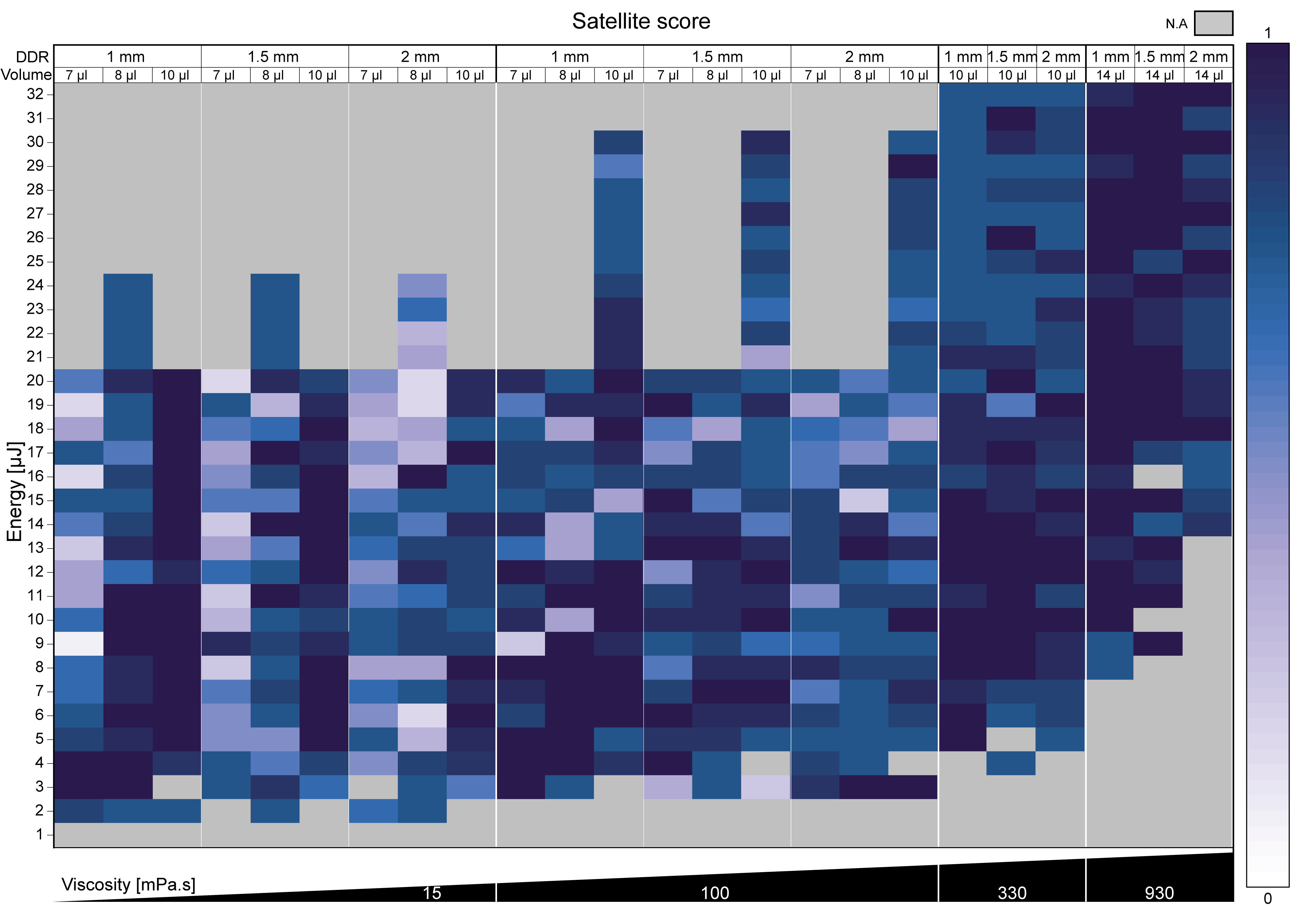


Supplementary Figure 2d Satellite score polymeric solution.

We have mapped the coefficient of variation of droplet diameter in order to reflect the repeatability of the findings.


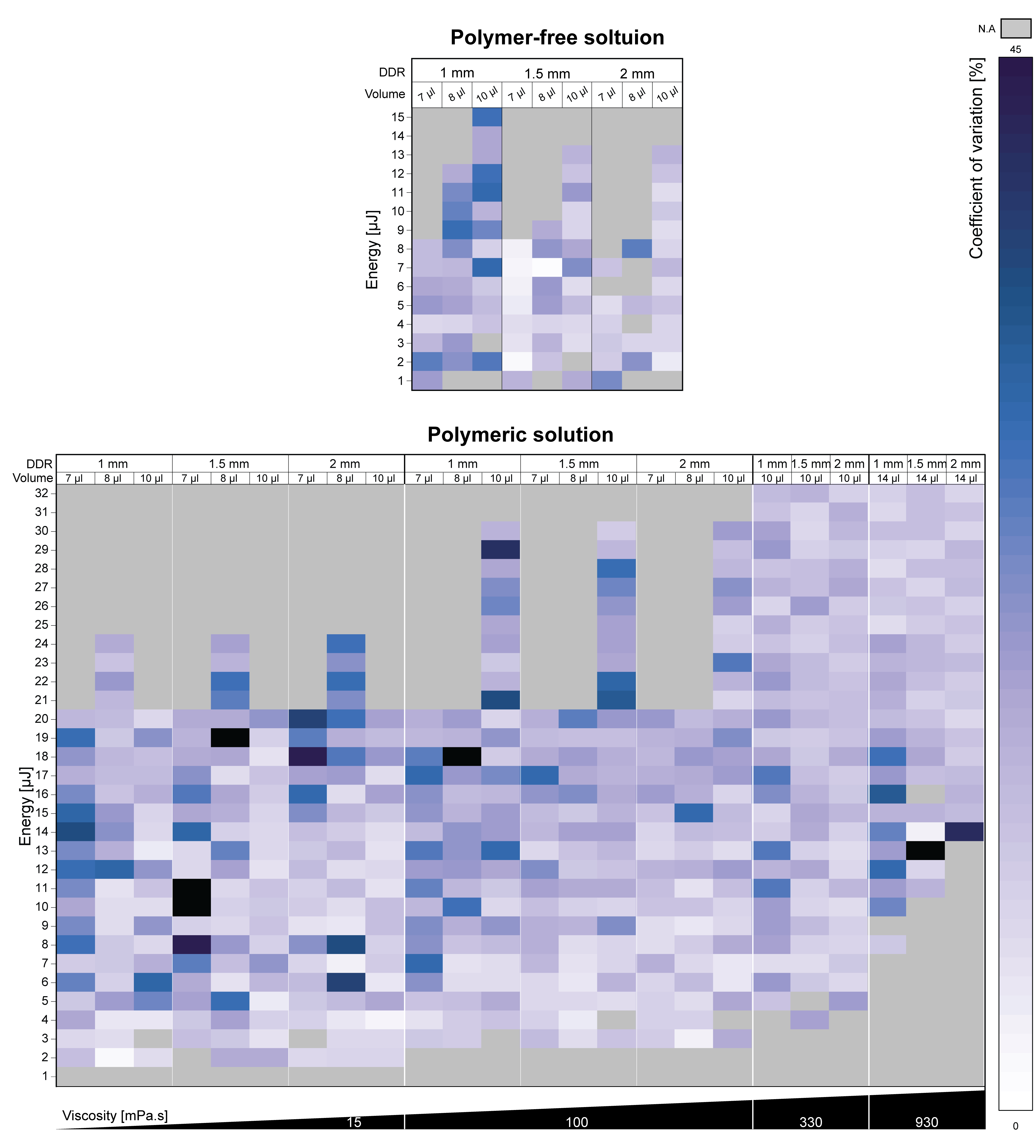


Supplementary Figure 3 Coefficient of variation of diameters for both polymer-free and polymeric solutions.

1. **Viscosity measurements - polymeric solutions**

The polymer solution consisted of alginate diluted at various concentration within a 0.1% BSA in PBS solution. The viscosity measurements are included below, measured with a cup setup (Anton Paar Physica MCR 301) at increasing shear rates from 0.01 to 100 s^−1^ followed by an overtime measurement (120 s) at a constant shear rate of 10 s^−1^.


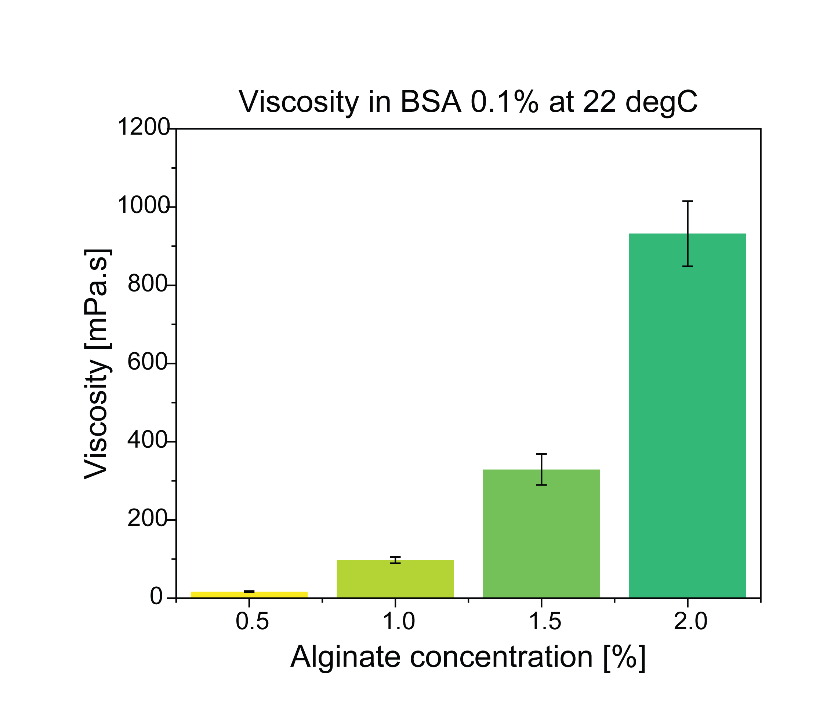


Supplementary Figure 4 Viscosity measurements of the polymeric solutions

1. **Viability after single-cell bioprinting**

NIH 3T3 fibroblasts were printed as single-cell containing droplets at 5 µJ, 1 mm DDR, 10 µL loading volume onto either a collagen type I (rat tail, 2.6 mg/mL) sheet or directly on a plastic tissue culture dish. The control consisted of cells seeded onto either substrate. Viability was assessed at day 1, 2, 3 and 7 post printing. While cells did not survive the printing on plastic, viability remained high for the collagen condition. Results demonstrate the necessity to include a cushioning layer to maintain cellular integrity and viability, as discussed in the main manuscript.


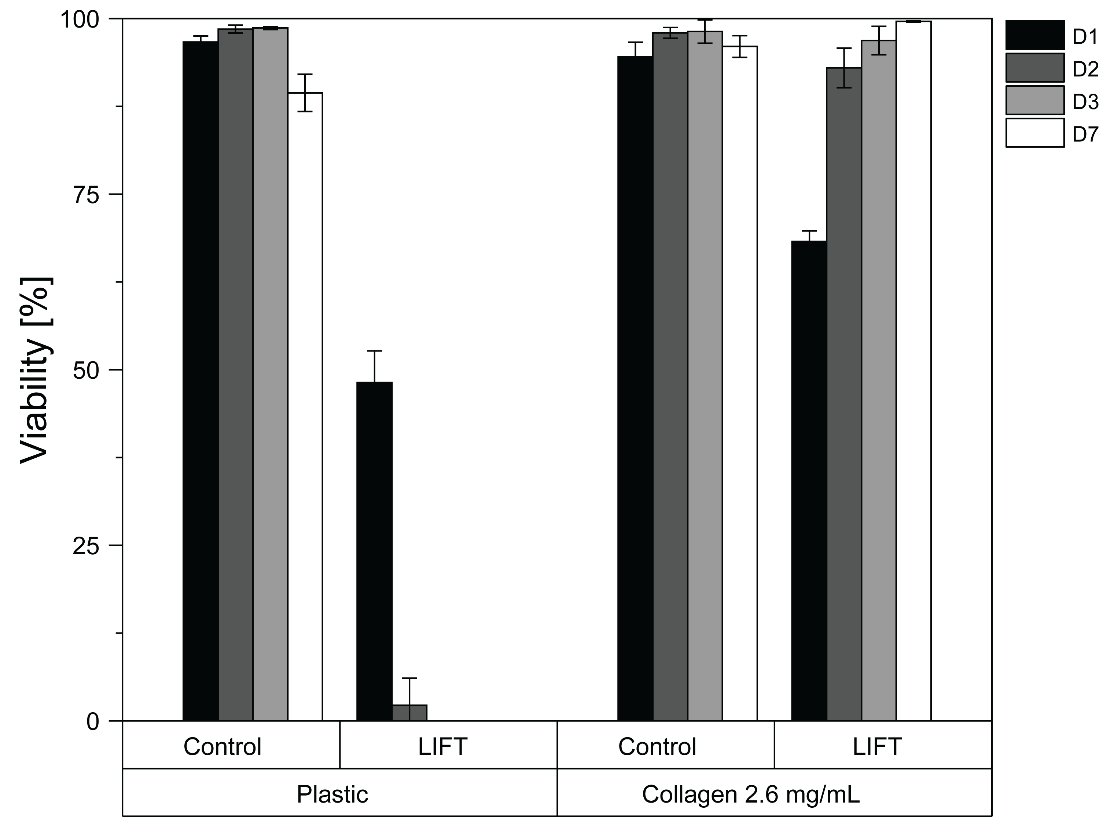


Supplementary Figure 5 Viability of NIH 3T3 fibroblasts printed as single cells at 5 µJ, 10 µL, 1 mm DDR on plastic or a collagen type I layer. Control designates cells seeded on either substrate.

1. **Droplet volumes originating from low versus high cell concentration bioinks**

Following the same methodology described in Figure 3 of the main manuscript, droplet volume was assessed over a broader range of initial ink concentration. The screening of n=29 inks with cell concentrations ranging from 11 to 50 × 10⁶ cells/mL and n=48 droplets per condition was performed. The droplet diameter was assessed at 531 ± 190 pL on average.


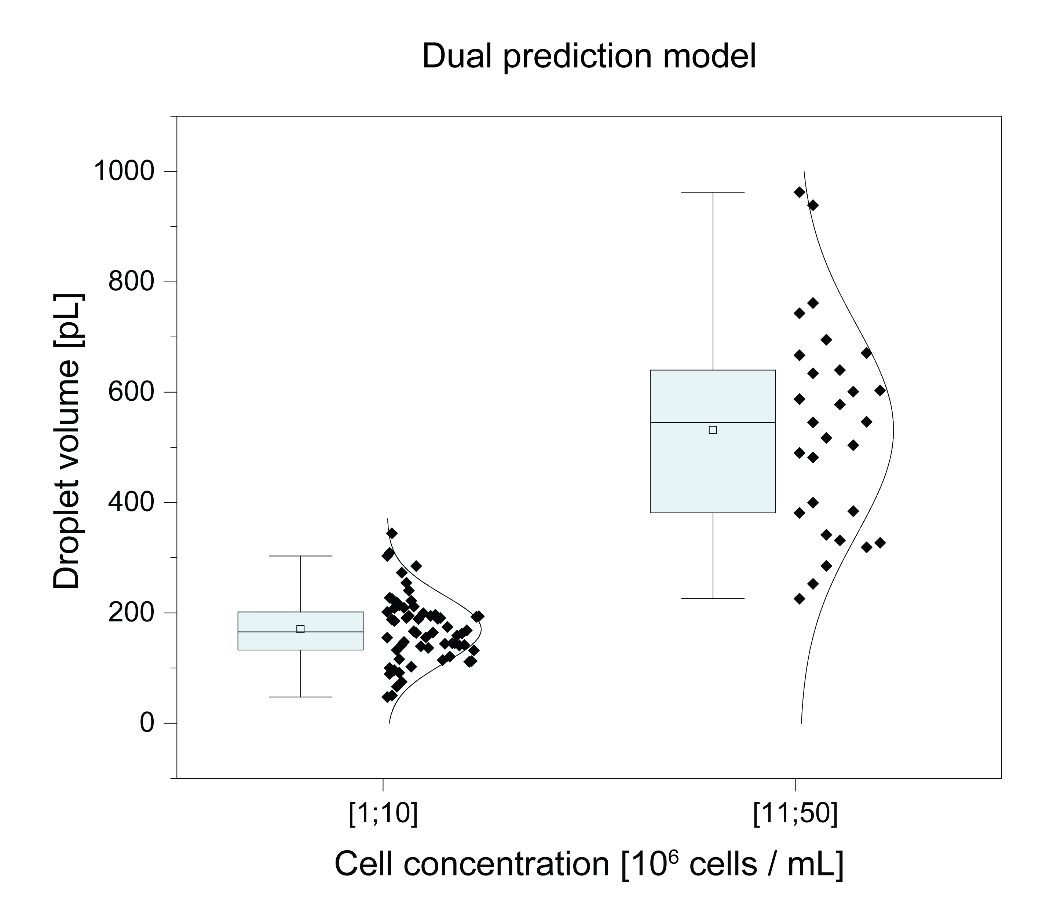


Supplementary Figure 6 Droplet volume estimation depending on cell concentration.

1. **Cell viability post-printing at high cell densities**


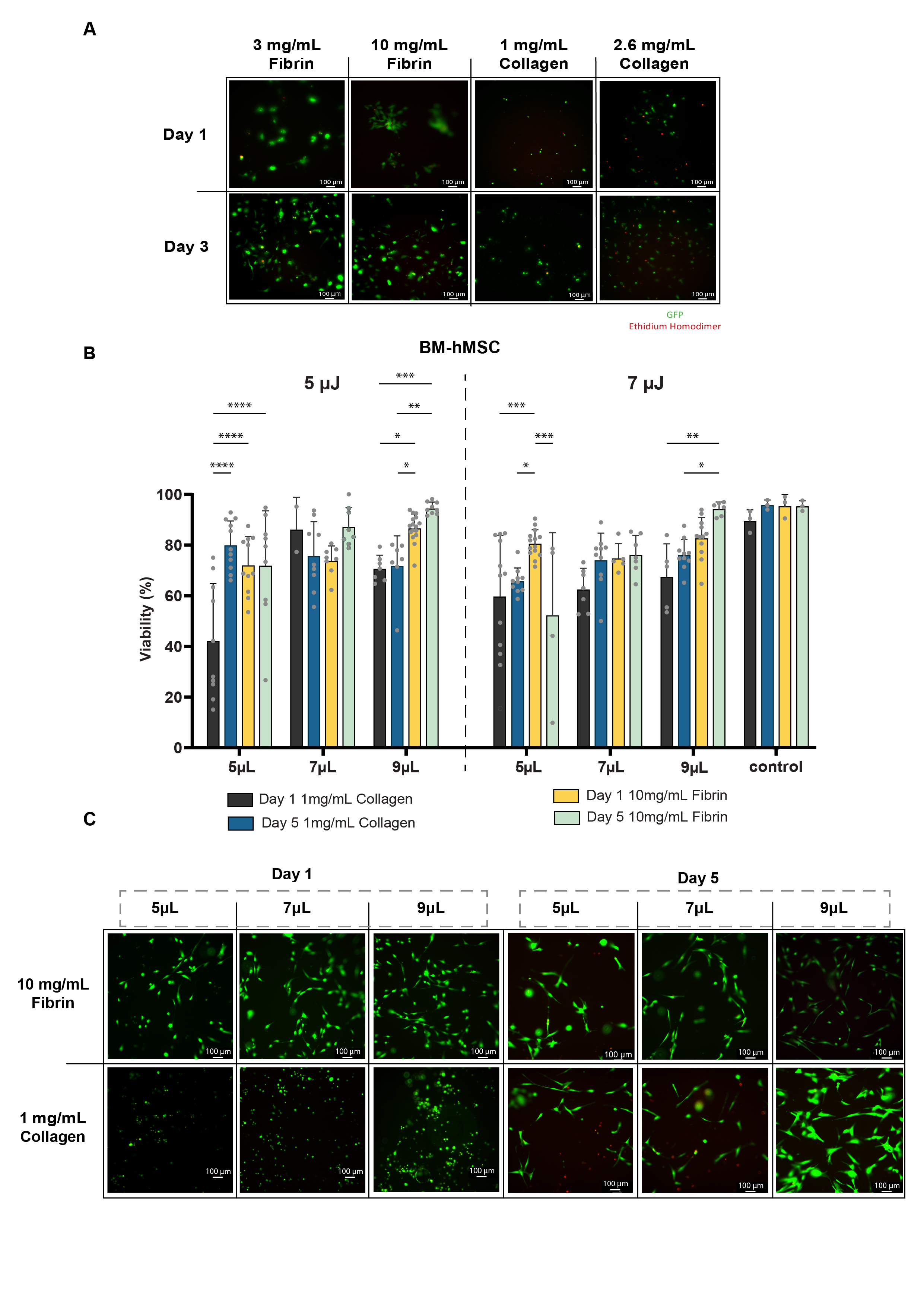


*Supplementary Figure 7. Cell viability. A) Representative images of HUVEC-GFP 30 × 10^6^ cells/mL suspended in complete EGM®-2 medium and LIFT-printed at laser energy of 7 µJ, DDR of 1.5mm, and application volume of 7 µL. Receiver compositions: 3 and 10 mg/mL Fibrin, and 1 and 2.6 mg/mL collagen type I. Images taken on days 1 and 3 after dead staining (GFP in green, Ethidium Homodimer in Red) using a Thunder microscope (Leica, Germany). Scale bar = 100 µm. B) Representative images of BM-hMSC 30 × 10^6^ cells/mL suspended in complete MSC expansion medium and LIFT printed at laser energies of 5 µJ and 7 µJ, DDR of 1.5 mm, and application volumes of 5, 7 and 9 µL. Images taken on days 1 and 5 after live and dead staining (Calcein-AM and Ethidium Homodimer) using a Thunder microscope (Leica, Germany) and analysed via Fiji (ImageJ 1.8.0) for particle analysis. Quantification via GraphPad 10.1 Prism. Sample size displayed in graph. C) Representative images of BM-hMSC LIFT-prints with Calcein-AM (green) and Ethidium Homodimer (red). Scale bar = 100 µm.*

1. **Pattern density study**

**
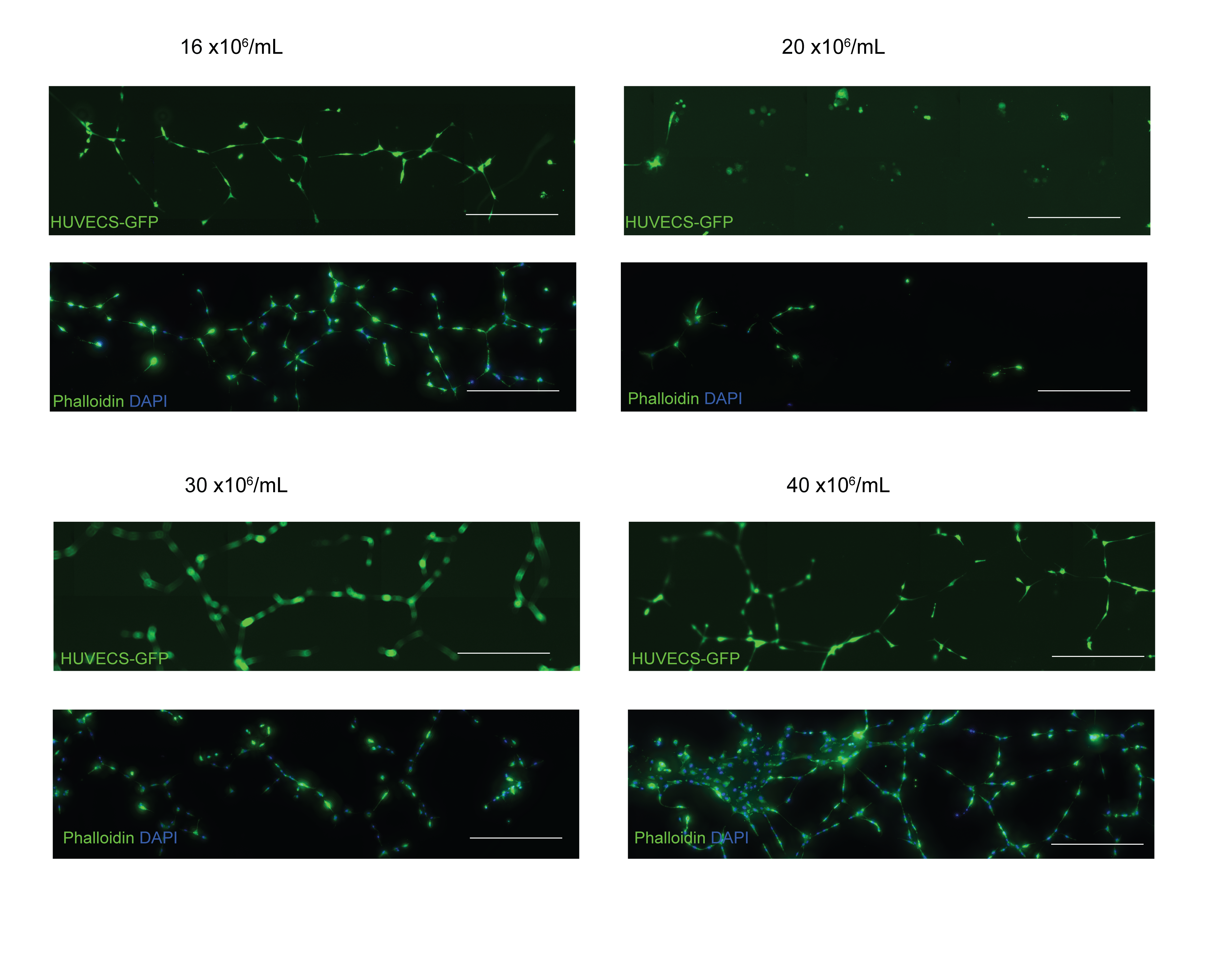
**

*Supplementary Figure 8. HUVECS-GFP on Matrigel 2 days post-LIFT. Pictures of two different biological replicates per conditions (focus on one field of view). Scale bar indicates 500 µm.*

1. **BM-hMSC and HUVEC-GFP co-culture**


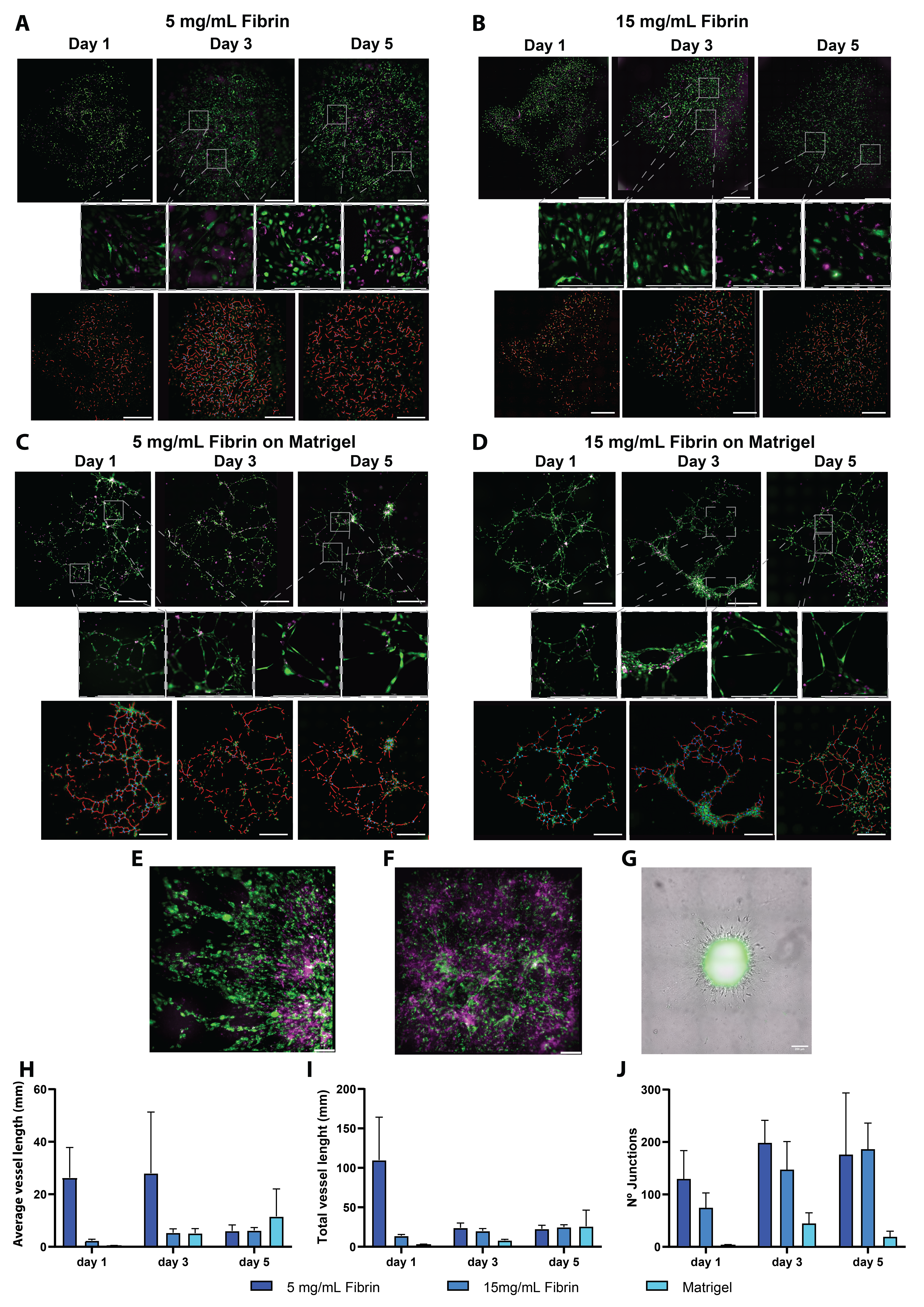


*Supplementary Figure 9. Establishing co-cultures via LIFT with high spatial complexity. A-D) HUVEC-GFP (green) and BM-hMSC (magenta) LIFT printed under four donor/receiver conditions: A) 5 mg/mL fibrinogen donor on 5 mg/mL fibrin. B) 15 mg/mL fibrinogen donor on 15 mg/mL fibrin receiver. C) 5 mg/mL fibrinogen donor on Matrigel supplemented with thrombin. D) 15 mg/mL fibrinogen donor on Matrigel supplemented with thrombin. All Images were acquired on a Thunder microscope (Leica microsystems, Germany) at days 1, 3 and 5 post-LIFT printing. Each panel is followed by corresponding micrographs of the indicated regions and AngioTool overlays used for vessel network quantification. Scale bars = 1mm. E-G) Cast controls: cell suspension in complete EGM-2 medium seeded respectively on E) 5 mg/mL Fibrin, F) 15 mg/mL Fibrin, and G) Matrigel receivers. Representative images taken on day 3 of culture using a Thunder microscope (Leica microsystems, Germany). Scale bars = 200 µm. H-J) Quantification via AngioTool of H) average vessel length, I) total vessel length, and J) number of junctions. n=4 control samples per quantified condition.* *All prints were performed at laser energy of 7 µJ, application volume of 7 µL and DDR of 1.5mm, with a total cell concentration of 30 × 10⁶ cells/mL in a 3:1 ratio of HUVEC-GFP to BM-hMSC.*
